# Emissions Footprints of Agriculture Around the World 1970–2020: Decreasing Land Conversion, Regional Exceptions and Increasing Management Intensity

**DOI:** 10.1111/gcb.70528

**Published:** 2025-10-02

**Authors:** C. A. Adlan, S. V. Hanssen, H. Luchtenbelt, C. Hendriks, J. C. Doelman, E. Stehfest, B. Wicke

**Affiliations:** ^1^ Department of Environmental Science Faculty of Science, Radboud University Nijmegen the Netherlands; ^2^ PBL Netherlands Environmental Assessment Agency The Hague the Netherlands

**Keywords:** agricultural crop production, agricultural management, emission intensity, emissions footprint, land‐use change

## Abstract

Land‐use change (LUC) and agricultural management associated with crop production are responsible for 13% of global anthropogenic greenhouse gas emissions. However, the quantification of such land use emissions for specific crops remains incomplete due to the exclusion of important emission sources and limited geographic or crop coverage in previous studies. In this paper, we derive global spatially explicit land use emissions from LUC and agricultural management over the period of 1970 to 2020 and attribute them to 16 agricultural crop types and pastureland for livestock production, using the IMAGE‐Land and LPJmL 4.0 models. Our results show 210 GtCO_2_‐eq were emitted over these 50 years; 69% of which were from LUC and 31% from agricultural management. To analyze trade‐offs between emissions and productivity, we generate emissions footprints (tCO_2_‐eq/ha) and emission intensities (tCO_2_‐eq/ton) per crop. Here, we define three clusters of crop‐region pairings that help prioritize measures for future reduction in land use emissions from agricultural crops: (i) high‐footprint, efficient land use (e.g., palm oil in Indonesia) where limiting conversion of high‐carbon stock area has first priority, (ii) low‐emission, inefficient land use (e.g., tropical cereals in Western Africa) where the focus should be on improved agricultural management to increase yields and thereby also minimize the need for land expansion, and (iii) moderate‐footprint and moderately efficient land use (e.g., soybeans in Brazil and rice in Southeast Asia) where a combination of agricultural management improvements and restricting land conversion can help most.

## Graphical abstract

Land‐use change and agricultural practices to produce crops cause 13% of GHG emissions, yet a comprehensive global picture of which crops and regions are responsible is still lacking. Using the IMAGE‐LPJmL model framework, we determined spatially explicit agricultural land use emissions worldwide between 1970 and 2020, attributing them at the grid level to 16 crop types and pasture used for livestock. This approach enabled us to calculate crop footprints (in tCO_2_‐eq/ha) and emission intensities (in tCO_2_‐eq/t crop) and explore trade‐offs between emissions and productivity. Based on these insights, we identify crop–region clusters with different priorities for reducing future agricultural emissions.

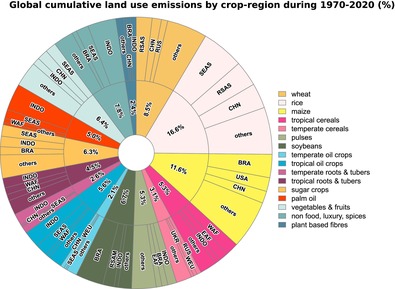

## 1 Introduction

Food is responsible for 26% of global anthropogenic GHG emissions (13.7 GtCO_2_‐eq), with half of these emissions stemming from land use change (LUC) (e.g., deforestation for conversion to agriculture) and agricultural management (e.g., fertilizer application, rice cultivation, and agricultural waste burning), which are collectively referred to as land use emissions (Poore and Nemecek 2018). Another 30% is caused by livestock production (e.g., enteric fermentation, manure and pasture management), and the remaining emissions are from supply chain activities such as food processing, transport, packaging, and retail. While food production is essential for humanity, the climate change impacts of agriculture require action to reduce emissions and align with the goals set in the Paris Agreement (Verschuuren 2016; Leahy et al. 2020). In order to shape mitigation policy, it is essential to understand how different agricultural crops contribute to land use emissions and how this has varied over time and across regions.

Spatially and temporally specific analysis of land use emissions can help shape policy in this regard in several ways. Information on region‐specific land use emissions attributed to specific crops (‘crop emissions’) allows producers to assess the environmental impacts of their products and define strategies to reduce them (Halpern et al. 2022; Vera et al. 2020). It also allows regional and national government actors to identify hotspots of emissions and mitigation efforts specific to their region or country and crops (Vergé et al. 2007). Furthermore, spatially explicit crop emissions show the geographic distribution of emissions, especially from LUC, and allow for the comparison across different countries or regions (Carlson et al. 2017). They also provide a better understanding of the sources and drivers of emissions (Hong et al. 2021; Pendrill et al. 2019), including across agricultural systems and types of producers (i.e., large‐scale companies and smallholders) (Abood et al. 2015; Austin et al. 2019). This information can help local decision‐makers to improve agricultural and land management practices (Lam, Chatterton, et al. 2021).

Various previous studies have employed detailed spatially explicit attribution methods, but they have often been limited in either geographical coverage or selection of crops (Austin et al. 2019; Garofalo et al. 2022). Spatially explicit land use emissions have also been analyzed at a global level, but studies such as Hong et al. (2021) have attributed emissions to crops at a country level and do not capture higher resolution spatial variation of crop‐specific emissions. In addition, existing global datasets of land use emissions footprints of crops (e.g., Agri‐footprint, Salim et al. 2024) rely on statistical analyses of FAOSTAT at the country level for the attribution, which masks the spatial variation of crop emissions. Yet, these databases are widely used by researchers in academia, business, and consultancy for the life cycle assessment of agricultural products and supply chains. These databases could be further improved by incorporating spatially explicit perspectives of land use emissions associated with crop production (Flynn et al. 2012).

In this study, we derive global spatially explicit land use emissions from agriculture over the period of 1970–2020 and attribute them to 16 agricultural crop types as well as pastureland for livestock production. We include CO_2_, N_2_O, and CH_4_ emissions from both LUC and agricultural management. We then generate emissions footprints (tCO_2_‐eq/ha) per crop type to understand the emissions of expanding agricultural land, and crop emission intensities (tCO_2_‐eq/ton yield) to assess the environmental efficiency of crop production across regions. We also provide further analysis to understand the trade‐offs between crop emissions and production.

## 2 Materials and Methods

**FIGURE 1:**
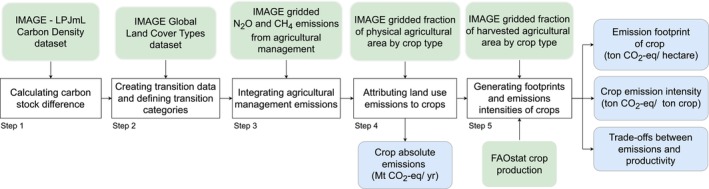
Main steps of this study (white boxes), input datasets (green boxes) and outputs (blue boxes).

This study assesses spatially and temporally specific agricultural land use emissions at a global level and attributes these emissions to agricultural crops and pasture. We combine several datasets on emissions from LUC and agricultural management derived from the IMAGE Integrated Assessment Model (IAM) framework 3.3 (Vuuren, Stehfest, Berg, et al. 2022). The current approach builds on historic land use and LUC dynamics over time and associated emissions. Our analysis consists of five main steps, as shown in Figure 1 and described in Sections 2.2, 2.6. The detailed descriptions of these steps are presented in Supporting Methods S1. Section A.

### 2.1 Model and Datasets Overview

The analysis builds on the results from two submodels of the IMAGE IAM 3.3 framework: IMAGE‐Land and LPJmL 4.0. IMAGE is an integrated assessment model (IAM) that projects global environmental impacts resulting from shifts in human activity under different scenarios (Stehfest et al. 2014). LPJmL is a long‐established dynamic global vegetation model (Müller et al. 2016), and it is fully coupled with, and an integral part of, IMAGE (Schaphoff et al. 2018). The IMAGE land‐use allocation model simulates land‐use dynamics and determines new agricultural land expansion (Doelman et al. 2018; Stehfest et al. 2014). Historical cropland and grazing areas are derived from the HYDE database (Klein Goldewijk et al. 2017), which is calibrated with FAO agricultural area (FAOSTAT 2024). Using these gridded land‐use dynamics, LPJmL simulates vegetation dynamics (turnover, mortality, competition for light and water, etc.) based on the concept of plant functional types (PFTs) defined by their biophysical characteristics (Müller et al. 2016; Schaphoff et al. 2018). These include natural vegetation, grassland, and crop PFTs.

Land use in IMAGE is represented at 5 arc‐min grid, which is downscaled to 30 arc‐min for coupling purposes with LPJmL (Doelman et al. 2018). Within this coupling, LPJmL computes daily weather variables from IMAGE's monthly climate data and operates fully dynamically at the annual time steps. Carbon dynamics are computed under land use change, CO_2_ fertilization, and climate change (Müller et al. 2016). Climate change is simulated via gridded temperature and precipitation derived from the internally coupled MAGICC model (Meinshausen et al. 2011). It is combined with spatial patterns from CMIP5 (AR5) climate model output using pattern scaling. A 30‐year average is used with interannual variability within LPJmL. These climate inputs, along with historical land use, are provided to LPJmL, which dynamically simulates vegetation productivity, soil processes, and carbon stock at 0.5° resolution. This resulted in a change in carbon sink capacity under climate change, which is reflected in the modeled carbon density at 5 arc‐min resolution.

Additionally, IMAGE determines the spatial distribution of irrigated and rainfed cropland area at 5 arc‐min. The potential yields of 16 crop categories (see Section 2.5) and grassland productivity are calculated by LPJmL at 30 arc‐min based on climate, hydrological, and soil properties. To convert potential yields to actual yields, a management factor (MF) is applied that calibrates outcomes per socio‐economic region on historic FAOSTAT. The management factors include both management intensity and harvest intensity and are part of the IMAGE calibration procedure (Doelman et al. 2022; Stehfest et al. 2014). Different spatial representations of irrigated and rainfed cropland, yields, and regional crop productivity require an upscaling and downscaling mechanism for exchanging purposes. The aggregated yield is compared to the regional demand for crops and grassland production. An iterative process is applied to allocate crop fractions in agricultural land use until the yield meets the required regional production. If calculated yields are insufficient to meet regional demand, agricultural areas are expanded into natural land. Conversely, if yields exceed demand, some agricultural land is abandoned (Vuuren, Stehfest, Elzen, et al. 2022).

In this study, we use gridded datasets on carbon density, land use, and agricultural land use per crop type and pastureland. These datasets have 5‐year temporal resolution from 1970 to 2020 (with 5‐year moving average) at 5 arc‐min spatial resolution. A 5‐year moving average is the default mechanism in IMAGE, aiming to capture the inertia inherent in socio‐economic and environmental processes, as IMAGE interacts with and is coupled to various internal models representing the Human and Earth systems within the IMAGE IAM framework.

### 2.2 Calculating Carbon Stock Losses

To determine LUC emissions, we calculated carbon stock loss from land use transitions. We generated carbon stock data at the grid level by multiplying carbon density (Mg carbon/km^2^) in LPJmL with the grid area. For this study, we consider only aboveground biomass pools (stems, branches, leaves) as this is the main (instant) change in biomass due to LUC. We calculated the change in total aboveground carbon stock over each 5‐year period. Soil carbon pools were excluded from the analysis due to their slow flux, which introduces an uncertainty when assigning these emissions to crops in a specific year.

### 2.3 Creating Transition Data and Defining Transition Categories

To track the dynamics of LUC, we created a transition dataset documenting individual LUC at the grid level during 1970–2020 based on the land use dataset. From 20 land use classes, we identified 103 unique land use transitions (Table S1), capturing the changes between different land use types over time. These transitions were further aggregated into six general transition categories: (1) Natural vegetation transitions, (2) Natural vegetation to agriculture, (3) Natural vegetation to biofuel, (4) Natural vegetation to extensive grassland, (5) Agricultural transitions, (6) Land abandonment to natural vegetation. Subsequently, these land use transitions were associated with carbon stock differences determined in the previous step. This was feasible due to the identical spatial and temporal resolution of both datasets. This allowed the classification of particular land use transitions for each time step as either emission or sequestration events, as only the emission events were used for the attribution step. When attributing emissions to crops, we only included two classes of transition emission categories; that is, natural vegetation to agriculture and agricultural transitions. We focused on these two categories because they are the sources of agricultural crop emissions.

### 2.4 Integrating Agricultural Management Emissions

In this step, we generated total land use emissions by first calculating non‐CO_2_ LUC emissions and emissions from agricultural management at the grid level and then combining these with LUC emissions from the previous step (Section 2.3). We utilized seven gridded emission source datasets related to non‐CO_2_ LUC emissions and both CO_2_ and non‐CO_2_ emissions from agricultural management (all obtained from: Doelman et al. 2018; Vuuren, Stehfest, Elzen, et al. 2022): (1) N_2_O from land clearing, (2) CO_2_, N_2_O, and CH_4_ from peatland drainage, (3) N_2_O from manure application on cropland, (4) N_2_O from agricultural residues, (5) N_2_O from synthetic fertilizers application on cropland, (6) N_2_O and CH_4_ from agricultural waste burning, and (7) CH_4_ from wetland rice cultivation. Extended descriptions of agricultural management emissions are provided in Supporting Information A3.

### 2.5 Attributing Land Use Emissions to Agriculture Crops

To analyze the total GHG emissions associated with the land use and cultivation of crops, we determined total crop‐specific absolute emissions at the grid level. The total LUC emissions (Section 2.3), including datasets 1–3 (Section 2.4), were attributed to different crops produced in a grid cell proportional to the increase in crop area fraction, using a dataset on the fraction of agricultural land by crop type dataset from the IMAGE model. Dataset 4 was attributed to crops in proportion to their aboveground agricultural residue, while datasets 5–7 are already modeled as crop‐specific datasets; hence, they did not require attribution. The crop types include 16 crop categories, including wheat, rice, maize, tropical cereals, temperate cereals, pulses, soybeans, temperate oil crops, tropical oil crops, temperate roots and tubers, tropical roots and tubers, sugar crops, palm oil, vegetables and fruits, non‐food and luxury and spices, plant‐based fibers, as well as one non‐crop category; that is, pastureland.

### 2.6 Generating Emissions Footprints and Emission Intensities of Crops and Defining Relationship With Crop Production

To analyze land use emissions in more detail, we looked at two types of metrics: emission footprints, which express LUC emissions per hectare of cropland (in tCO_2_/ha), and emission intensities, which express emissions per ton of product (in tCO_2_‐eq/ton crop) and incorporate emissions from both LUC and agricultural management. Both metrics are determined for 16 crops at the regional level and calculated for each 5‐year step. For comparison across crops, we then averaged the value over the entire accounting period.

We determined two varieties of emissions footprints of crops; that is, marginal (newly converted crop area) and total (total crop area). For the marginal crop footprint (tCO_2_/ha), we aggregated the grid‐level emissions of crop‐specific natural vegetation conversion to agriculture to the 26 IMAGE regions (see Figure S1), using SEDAC gridded country boundary as an identifier (CIESIN 2018). We then aggregated the newly converted area from natural vegetation to agriculture transitions (Section 2.3) from the grid level to the regional level. Lastly, at the regional level, we then divided the natural vegetation to agriculture emissions by the total newly converted cropland area, resulting in a marginal crop emissions footprint of crops (tCO_2_/ha). The fact that such marginal footprints can be derived is a key advantage of our spatially explicit approach. For the total emissions footprint of crops (tCO_2_‐eq/ha), we calculated the total crop area by combining the existing crop area from the previous time step with newly established cropland. We divided the total land use emissions, comprised of LUC and agricultural management emissions, by total crop area.

We determined the crop emission intensity (tCO_2_‐eq/ton) at the regional level to understand the efficiency of crop production in terms of GHG emissions across regions. We do so by dividing the total land use emissions by the total crop primary production. Using data from the FAOSTAT dataset, we calculated total crop production in three steps. First, to align with the IMAGE's crop categorization, we aggregated primary production data from FAOSTAT into IMAGE's corresponding crop category (Table S2). Second, we aggregated country production data to the regional level. Third, we applied a 5‐year moving average to the FAOSTAT data to ensure consistency with the emissions data, smooth interannual variability, and highlight general trends rather than short‐term anomalies, such as yield drops during droughts. This method avoids attributing all emissions to a single crop year. We assume that typical crop rotation patterns are represented within each five‐year window, allowing for a more balanced and implicitly apportioned attribution of emissions across crop rotation. Historical IMAGE data are calibrated to FAOSTAT by applying two management factors ensuring consistency between IMAGE and FAOSTAT. First, harvest intensity in IMAGE indicates how often a physical crop area is harvested annually (value of 2 for double cropping or 0.5 for biennial fallow). It is used as a calibration factor to convert crop physical area into harvested area, aligning with the FAOSTAT reported harvested area. Second, management intensity is applied to scale potential yields (at 5‐arcminute resolution) to actual yields using a calibration factor that ensures consistency with regional FAO crop yield statistics.

We also analyzed the relationship between emissions and crop production, decomposing the factors determining emission intensity of crops. To do so, we plotted the total emissions footprint of crops against land requirements for crop production (the inverse of crop yield; hectares/ton). We calculated the land requirement at the regional level by summing harvested area per crop category at the regional level and dividing it by the sum of crop production from the second step. We calculated the land requirement for each 5‐year step and then averaged it across our accounting period. Crop emission intensity can be inferred from the multiplication of the total emissions footprint and the corresponding land requirement. To highlight the comparison between important regions at the global level, all results presented below were aggregated to 26 regions (PBL 2022).

## 3 Results & Discussion

### 3.1 Land Use Patterns

**FIGURE 2:**
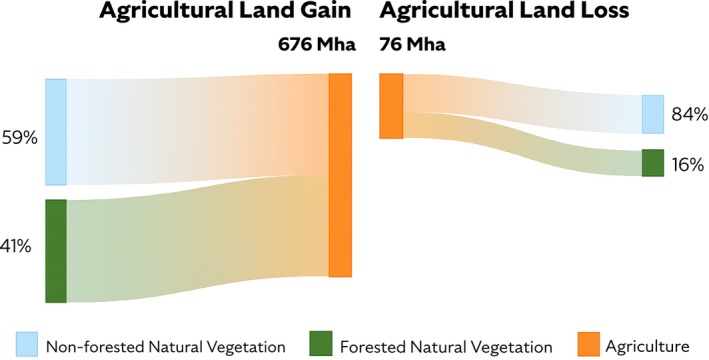
Global land‐use transitions over 50 years (1970–2020). Detailed land use transitions per region are presented in Figure S2.

Global land use patterns have undergone significant shifts over the past 50 years. Our results indicate that 676 Mha of natural vegetation have been replaced by agricultural land (Figure 2). At the same time, 76 Mha of agricultural land were abandoned, resulting in the conversion to steppe, scrubland, or forest. Overall, the conversion of natural vegetation to agriculture represents a 20% increase in the total area used for agriculture since 1970. Non‐forested natural vegetation, such as savannas, steppes, and scrublands, underwent the most significant area conversions, collectively accounting for 59% of the total natural vegetation area converted to agriculture. Forested natural vegetation, such as tropical forests and tropical woodlands, experienced a combined 16% conversion to agriculture. Note that conversion to intensively grazed grassland (pastureland) is included in the agricultural land category and accounted for 56% of natural vegetation conversion, while crop cultivation contributed 44%. Among the 16 crop types considered in our study, maize, soybeans, tropical cereals, and wheat saw the largest increase in crop area, collectively accounting for 39% of the total conversion to cropland. Regions such as China, Brazil, the rest of South America, and Eastern and Western Africa have witnessed the largest absolute agricultural expansion with a total cumulative of 440 Mha to meet increasing food demands. Our results show 62% of natural vegetation conversion to agriculture occurred in tropical regions, compared to 38% in non‐tropical regions (Table S3–S6).

### 3.2 Land Use Emissions

Global cumulative land use emissions over 50 years were 210 GtCO_2_‐eq. LUC contributed 146 GtCO_2_‐equation (69%) of these emissions. Specifically, emissions from converting natural vegetation to agriculture (cropland and pasture) played an important role, contributing 65% of all land use emissions. This is because these transitions involve the largest reductions in carbon stocks. Agricultural transitions; that is, changing from one crop type to another and intensive pasture to crop vice versa, caused only 3% of emissions, as carbon stocks are typically not altered much. The remaining 31% (64 GtCO_2_‐eq) of land use emissions come from agricultural management emissions: 12% from drained peatlands, 8% from fertilizer application (7% from synthetic fertilizer application and 1% from manure application), 7% from wetland rice cultivation, 3% from agricultural residue decomposition, and 1% from agricultural waste burning.

**FIGURE 3:**
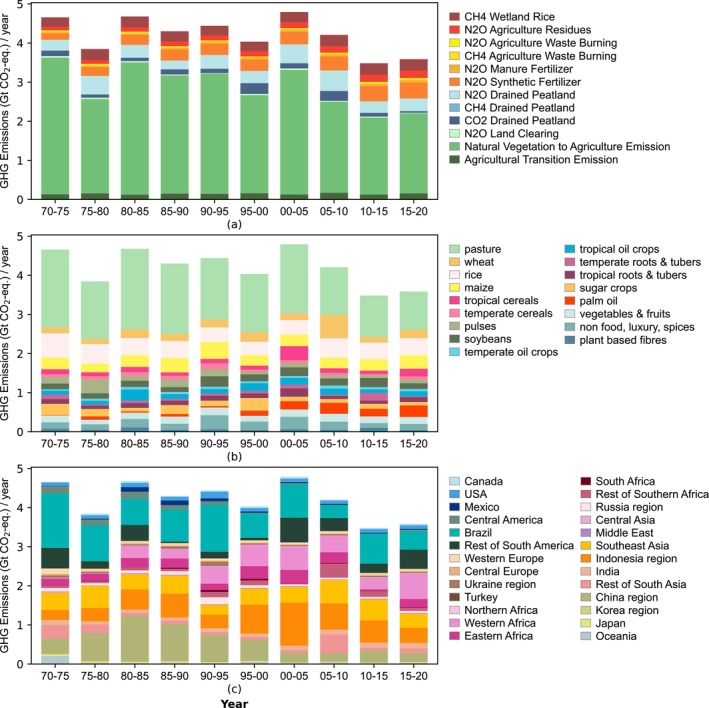
Average annual land‐use emissions differentiating: (a) LUC emissions and agricultural management emissions, (b) crop categories and pasture, and (c) countries and regions. Attributing only LUC emissions to crops and pasture and to regions is presented in Figure S4.

Land use emissions fluctuate over the years (Figure 3a) with the annual average ranging from 25% below to 3% above the 1970 level (4.7 GtCO_2_‐eq/year). On the whole, however, land use emissions from agriculture exhibit a decreasing trend. This is due to a decline in LUC emissions. Notable exceptions to this trend are increased LUC emissions of palm oil (+1766%), temperate oil crops (+73%), and tropical cereals (+44%) when comparing 2020 to 1970. Between 1970 and 2020, average annual LUC emissions decreased from 3.7 to 2.2 GtCO_2_‐eq while average annual agricultural management increased from 1 to 1.4 GtCO_2_‐eq. Our findings on LUC emissions from the conversion of natural vegetation to agriculture align with previous estimates of global LUC emissions to agriculture. Several studies have reported lower values of 1.68 ± 0.47 GtCO_2_/year during the 2009–2018 (Gasser et al. 2020) and 1.48 GtCO_2_/year during 2011–2020 (Houghton and Castanho 2023), while Pendrill et al. (2019) have found higher values of 2.60 GtCO_2_/year during 2010–2014. Our results, with emissions of 2.3, 2, and 2 GtCO_2_/year for 2005–2010, 2010–2015, and 2015–2020 respectively, fall within this range in the literature.

When comparing to the four bookkeeping model estimates included in the Global Carbon Budget 2024 project (Friedlingstein et al. 2025) and the National Greenhouse Gas Inventory (NGHGI) report values, we found that our emission estimates are consistently lower than those estimates, but that they show similar trends (see Figure S3a). The differences in magnitude reflect fundamental methodological differences in how IMAGE‐LPJmL, bookkeeping methods, and NGHGI calculate LUC emissions. These methods also vary substantially in the specific LUC and carbon density datasets employed (see Section 3.7). Based on Grassi et al. (2021) and Nabuurs et al. (2022), we discuss the conceptual differences in how global anthropogenic net CO_2_ emissions from land use are defined in different approaches (see Figure S3b).

Increasing agricultural management emissions reflect the expansion of agriculture (Figure 2) and a substantial rise in agricultural intensification practices like the increased use of fertilizers. The highest percentage increases in agricultural management emissions were observed for tropical oil crops (+193%), pulses (+179%), wheat (+157%), and soybeans (+107%). Additionally, the three crops that exhibit increases in LUC emissions (palm oil, temperate oil crops, and tropical cereals) also demonstrate substantial growth in agricultural emissions, mirroring their expanding crop areas. N_2_O emissions from applying synthetic fertilizer represent the largest increase in the agricultural management emissions category. Maize (+58 MtCO_2_‐eq), wheat (+37 MtCO_2_‐eq), rice (+33 MtCO_2_‐eq), and sugar crops (+32 MtCO_2_‐eq) exhibit the largest absolute changes in the annual average of synthetic fertilization emissions between 1970 and 2020. This suggests increased investment in the production of these crops to boost productivity, as these crops are not major contributors to (LUC) emissions. With less than half (42%–47%) of applied nitrogen fertilizers being absorbed by crops and the rest being emitted to air and water, improving farmers' knowledge and practices to achieve higher nitrogen use efficiency (NUE) is a key mitigation measure (Lassaletta et al. 2014; Li et al. 2025; Zhang et al. 2015).

Land‐use emissions are decomposed into 16 crop categories and one pastureland category (Figure 3b). Pastureland has consistently been the primary driver of land‐use emissions, contributing 27%–44% of average annual emissions over the accounting period. When considering only emissions from LUC, the contribution of pastureland even increases to 44%–58% (Figure S4). These large emissions associated with pastureland are caused by the conversion of 376 Mha of natural vegetation over 50 years, 40% of which was forested. This is primarily due to the pressure to expand the livestock production area, especially for the cattle industry. For cropland, the most significant crop categories are the three major staple cereals: maize, rice, and wheat, which together account for 17%–33% of average annual emissions. The sustained emissions associated with major staple cereals were largely driven by the growing food consumption of the rapidly growing human population (Schneider et al. 2011; Tilman et al. 2011), as these crops together provided 60% of the world caloric intake (Palacios‐Rojas et al. 2020). Other crops significant for emissions are soybeans, tropical oil crops, and non‐food and luxury and spices (i.e., rubber, cocoa), and contributed 3%–7%, 2%–6%, and 3%–8% to the annual land use emissions, respectively. While palm oil ranks only 11th out of 16 crop types in terms of cumulative emissions over 50 years (Table S7), it plays a more prominent role in more recent years. For example, in 2020, it ranks third only after rice and maize (Table S8).

We also decomposed the land‐use emissions into 26 regions (Figure 3c) and made comparisons between regions (Figure S5). Here we see that Brazil's emissions peaked during 1970–1995, when emissions were at record highs of 1.4 GtCO_2_‐eq/year in 1975. South America and Southeast Asia are also traditional hotspot regions, but both also exhibit large fluctuations in land‐use emissions over time. Emissions were high in the earlier years, decreased between 1990 and 2000, and then increased again thereafter. China reached emissions of up to 1.2 GtCO_2_‐eq/year between 1980 and 1985, but emissions later decreased towards 0.2 GtCO_2_‐eq/year. Developing regions such as Indonesia and Western Africa show significant growth in emissions in more recent decades. Indonesia's emissions increased from 0.1 to a peak of 1.1 GtCO_2_‐eq/year, while Western Africa's emissions rose from 0.02 to 0.7 GtCO_2_‐eq/year.

### 3.3 Crop‐Region Emissions

**FIGURE 4:**
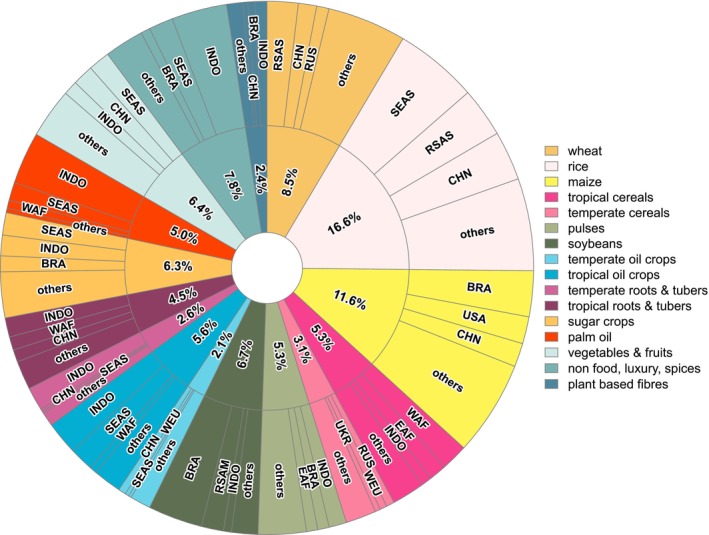
Global cumulative land use emissions by crop‐region in percent during 1970–2020, pasture is excluded because of much higher emissions that would not allow differentiating between other crops (figure with pasture included is presented in Figure S7). Only the three largest emitting regions per crops are shown, the remaining regions are classified as other.

We further analyzed cumulative land‐use emissions for all crop‐region pairings excluding pasture (390 unique pairings) (Figure 4; Table S7) and their temporal variation (Figure S6). The three crop‐region pairings with the highest emissions are rice in Southeast Asia (SEA), non‐food, luxury, and spices in Indonesia, and soybeans in Brazil with 5.1%, 3.4%, and 3.3% of cumulative emissions, respectively. Looking only at emissions for the most recent time step (2020), only rice in SEA (5.3% of total 2020 land use emissions) remains in the top three, topped by palm oil in Indonesia (6.4%) and followed by tropical cereals produced in West Africa (3.9%) (Table S8).

Among the 100 crop‐region pairings with the highest cumulative emissions (Table S7), two primary categories emerged. The first includes those crop‐region pairings with higher agricultural management and lower LUC emissions crops, such as maize in the USA, China, and Canada; wheat in the USA, Canada, India, and Europe; and sugar crops in SEA, Indonesia, and India. These crops are characterized by significant past LUC (prior to 1970) and limited suitable land for further expansion. These conditions drive intensification and result in higher agricultural management emissions. For instance, maize in the USA, the world's largest producer, has contributed 2.2 GtCO_2_‐eq in cumulative emissions, 93% of which stem from agricultural management and 56% of which are due to synthetic fertilizer use alone. Rice cultivation in various regions also falls into this category, with methane emissions from flooded fields contributing 61% of the total cumulative 22.2 GtCO_2_‐eq.

The second category includes crop‐region pairings with higher and often increasing LUC emissions and relatively lower agricultural management emissions. These crops are cultivated in regions with abundant suitable land with high carbon stocks, such as soybeans in Brazil and the rest of South America (RSAM), palm oil in the Indonesia region, tropical oil crops in SEA, and maize in Brazil. Our results show, for instance, that soybeans in Brazil were responsible for 4.4 GtCO_2_‐eq, 95% of which are caused by LUC (Table S7). While soybean is a primary driver of LUC in Brazil, they are also the region's most traded commodity, with its tropical forest loss correlated to an export‐oriented agricultural sector (DeFries et al. 2010; Garofalo et al. 2022; Song et al. 2021). Palm oil plantations in the Indonesia region contributed 4.2 GtCO_2_‐eq in cumulative emissions; 59% of these are caused by LUC. Our results show that 5.5 Mha of natural vegetation was converted to palm oil plantations, 92% of which originated from forested areas. The fact that these two crops are produced primarily for consumption in other countries points to the importance of engaging these importing countries and their financial support for limiting expansion into forested natural vegetation and increasing yields. Significant emissions from this category of crops also arise from maize cultivation in Central America and Western Africa, as well as from tropical cereals in Eastern and Western Africa. Although these crops are primarily cultivated for domestic consumption, low yields of existing cropland have driven cropland expansion, with LUC emissions accounting for over 94% of total emissions in each case.

### 3.4 Emissions Footprints of Crops

**FIGURE 5:**
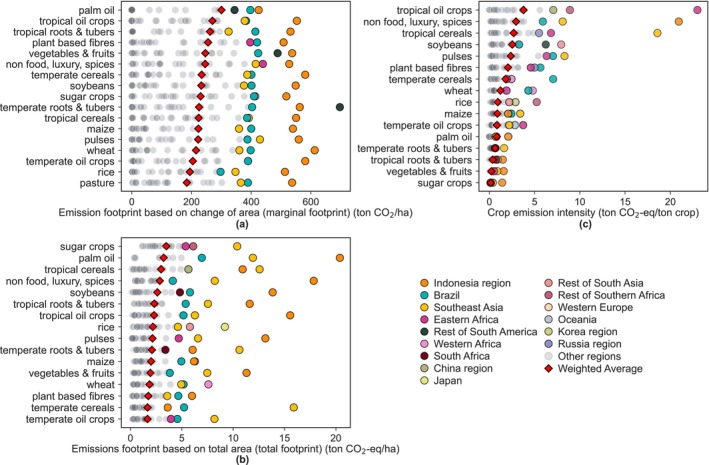
Average emissions footprints and emission intensities across 26 regions where (a) emissions footprint based on change of area (marginal crop footprint, tCO_2_/ha), (b) emissions footprint based on total area (total emissions footprint of crops, tCO_2_‐eq/ha), and (c) crop emission intensity (tCO_2_‐eq/ton). Top 3 emitter regions marked with colours, the rest with grey and the weighted average per crop in red diamond. The order of crops on the y‐axis is based on the weighted average emissions footprint/intensity.

We distinguish emissions footprints of crops values based on only the newly converted area (marginal footprints; Figure 5a) and on the total area (Figure 5b). These two approaches provide different insights. The marginal footprint reflects the direct emissions of expanding agricultural land and is particularly relevant for understanding the emissions risks of expanding cultivation. The total footprint assesses the overall emission impact of crops and provides a comprehensive picture of emissions from existing agricultural systems. In general, marginal footprints have much higher weighted average values (184–300 tCO_2_/ha) than the weighted average of total emissions footprints (1.6–3.5 tCO_2_‐eq/ha), and the order of highest emitting crops is different between the two approaches. Crops associated with high LUC, such as palm oil and tropical oil crops, typically rank high in the marginal footprint. In contrast, crops with extensive existing cropland with intensive management practices, such as rice and sugar crops, are likely to rank higher in the total footprint. These differences highlight the importance of distinguishing between the emissions associated with crop expansion (marginal footprint) and the shared responsibility for emissions from total crop production, which includes both existing and newly added cropland (total footprint).

Brazil, the Indonesia region, and SEA form the top emitting regions across crops for marginal crop footprint, as high carbon stock areas have been converted in these regions. As an example, palm oil in the Indonesia region has an average marginal footprint of 425 tCO_2_/ha. Other studies found that when a primary rainforest is replaced by a palm oil plantation, carbon emissions were estimated at 600–693 tCO_2_/ha (Danielsen et al. 2009; Guillaume et al. 2018; Kotowska et al. 2015; Saatchi et al. 2011). Our lower results stem from the fact that we average across various types of converted lands, including both higher carbon stock areas like tropical forests and lower carbon stock areas such as tropical savannas, regrowth forest timber, and tropical woodlands. Crops with high recent marginal footprints require immediate attention for reducing future risks of LUC, particularly limiting further conversion of natural vegetation with high carbon stocks.

### 3.5 Crop Emission Intensities

Crop emission intensities (tCO_2_‐eq/ton crop) show large variability across crop types, ranging from 0.004 tCO_2_‐eq/ton (temperate oil crops in the Indonesia region) to 23 tCO_2_‐eq/ton (tropical oil crops in Eastern Africa) (Figure 5c). Based on weighted average values, tropical oil crops exhibit the highest emission intensity among all crops at 3.8 tCO_2_‐eq/ton. The higher crop emission intensity suggests lower environmental efficiency of crop production, resulting from a combination of high LUC emissions and agricultural management emissions alongside low crop production. In contrast, sugar crops and palm oil have a relatively low emission intensities, at 0.1 and 0.8 tCO_2_‐eq/ton. Despite being the among the largest contributors to emissions, these crops exhibit higher productivity levels, emphasizing the complex trade‐offs between emissions and agricultural output (see Section 3.6). For Brazilian soybeans, we find emission intensities ranging from 0.1–8.3 tCO_2_‐eq/ton, with an average value of 3.2 tCO_2_‐eq/ton. This aligns with the wide variation reported within and across other studies, which range from 0.89 tCO_2_/ton during 2000–2010 (Persson et al. 2014) to 1.7 tCO_2_/ton for the period 1994–2014 (Lam, Sim, et al. 2021) or 0.13 to 29.4 tCO_2_‐eq/ton (with a national average of 0.69 tCO_2_‐eq/ton) for 2008–2010 (Escobar et al. 2020). We also compared emission intensities for Indonesian palm oil and SEA rice with values from other studies (Figure S8). Our estimates for palm oil (1.2–12.6 tCO_2_‐eq/ton, 1990–2020) fall within the previously reported range of 1.5–30 tCO_2_‐eq/ton, while our estimates for rice (0.9–2 tCO_2_‐eq/ton, 1990–2020) align with reported values of 0.8–3 tCO_2_‐eq/ton.

### 3.6 Trade‐Offs Between Emissions and Production

**FIGURE 6:**
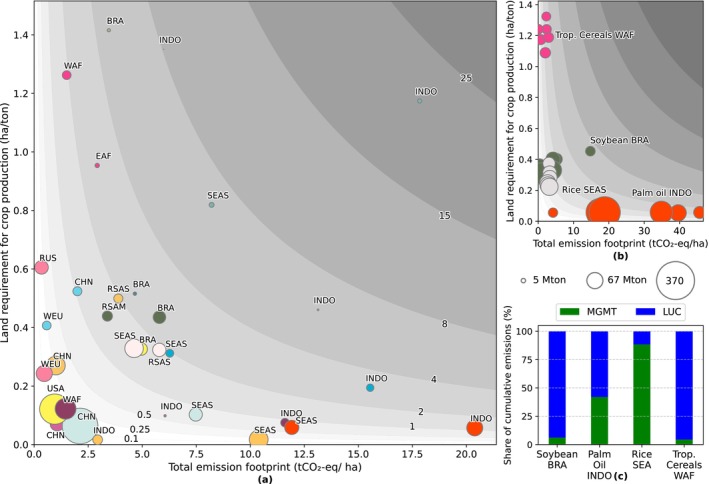
Relationship between total emissions footprint of crops (tCO_2_‐eq/ha) and land requirement for crop production (ha/ton, inverse of yield), with (a) average for two highest region emitters in each crop categories, (b) annual average variation for selected pairings from 1990 to 2020, and (c) shares of cumulative land‐use emissions from agricultural management (MGMT) and land use change (LUC) in percent for selected pairings during 1990–2020. For panel a and b, the background contours represent the emission intensity (tCO_2_‐eq/ton crops), the plot size indicates crop production, and colours represent crop types, following the same legend as in Figure 4.

Targeting specific crop‐region pairings for emission reduction is a complex task, given the trade‐offs between reducing emissions and maintaining crop production levels to meet food demand or ambitioned self‐sufficiency ratios in a certain region (Bennetzen et al. 2016). The marginal crop footprint of crops (tCO_2_/ha), total emissions footprint of crops (tCO_2_‐eq/ha), and emission intensity (tCO_2_‐eq/ton crops) indicators each provide insights on the most relevant crop‐region pairings in terms of emissions. Pairings that score high in one or more indicators represent the most important areas to focus on. Yet, using these three indicators separately is insufficient in assessing trade‐offs between emissions and crop production. Therefore, we illustrate how land requirements of crop production (ha/ton crop; that is, the inverse of yields) and total emissions footprints of crops (tCO_2_‐eq/ha) together constitute emission intensities of crops (tCO_2_‐eq/ton crop) and, when multiplied with production volumes, also determine overall emissions (Figure 6a). While crop‐region emission values fluctuate over time (Figure 6b), we present average values for simplicity. This approach also helps decompose emission intensity, as different crop‐region pairings may fall within the same range (see contour) yet reflect distinct circumstances with differing emissions reduction potential. We highlight four crop‐region pairings that have large crop production volumes and high cumulative (Figure 4) or recent (Figure S6) land‐use emissions: (1) soybeans in Brazil, (2) palm oil in the Indonesia region, (3) rice in Southeast Asia, and (4) tropical cereals in Western Africa. For these crop‐region pairings, we identify the largest sources of land‐use emissions (Figure 6c) and their regional contexts.

Our results show that the four selected crop‐region pairings fall into three clusters, defined by high‐to‐low emissions footprint and high‐to‐low land requirement. The first cluster is crops with high emissions footprints but efficient land use. Palm oil in Indonesia region is a prime example of this cluster. Palm oil production in this region increased almost tenfold, from 4.7 million tons in 1995 to 44.5 million tons in 2020 (FAOSTAT 2024). The growth in palm oil cultivation is largely driven by palm oil's versatility for use in a diverse applications and its high yields per hectare compared to other vegetable oils (Chiriacò et al. 2024; Parsons et al. 2020). This dramatic increase led to a twentyfold rise in annual average emissions from land use for palm oil, reaching 0.16 GtCO_2_‐eq/year by 2020. Over the entire evaluation period, LUC causes more than 50% of the emissions (Figure 6c). However, there is significant variation in LUC emissions, as shown by variation in the total footprint for individual time steps (Figure 6b). Gunarso et al. (2013) reported that 2.2 Mha of natural forest, including swamp forests and upland forests, were cleared for palm oil plantations in Indonesia and Papua New Guinea between 1990 and 2010. Our study found a similar loss of 2.3 Mha of tropical forests and woodlands in these regions over the same period. This cluster presents significant potential for emission reductions such as limiting high‐carbon stock area conversion and directing development toward low‐carbon stock areas, without compromising critical social and environmental values (Senior et al. 2015). In 2011, the Indonesian government enacted a moratorium on issuing new permits for large‐scale plantations in primary forests and peatlands (Pacheco et al. 2020). However, emissions in the region remained high over the following decade, reaching a total of 0.8 GtCO_2_‐eq during 2015–2020. Studies suggest the moratorium was ineffective due its exclusion of secondary forests, allowing land clearing for palm oil plantations to persist (Margono et al. 2014; Murdiyarso et al. 2011). Additionally, smallholders, still exempt from the regulation, have emerged as major LUC emissions contributors, surpassing large‐scale plantations as the leading deforestation driver (Austin et al. 2019). In addition, although palm oil is a high yielding crop, there is still significant room for improvements of yields, particularly for smallholder producers (Sugianto et al. 2025).

The second cluster is characterized by crop regions with low emissions but inefficient land use. This cluster includes tropical cereals in Western Africa (WAF). Millet and sorghum are important staple crops in WAF (Haggblade et al. 2012; Weltzien et al. 2018), particularly in the semi‐arid tropical region where the cultivation of other crops is generally limited. However, sorghum shows a persistent low productivity due to climate variability and low‐input farming systems (Adam et al. 2020; Macauley and Ramadjita 2015; Mason et al. 2015; Sultan et al. 2013). Despite low yields (see also its high land requirement in Figure 6b), increasing demand has driven expansion and the sorghum‐planted area increased from 5.6 to 12.2 Mha between 1981 and 2012 (Orr et al. 2021). Our results show that LUC emissions contributed 95% from a total of 2.1 GtCO_2_‐eq from 1970 to 2020. We found that 9.3 Mha of natural vegetation was converted for tropical cereals in WAF, with 7 Mha originating from non‐forested areas (64% savanna, 35% scrubland, and 1% steppe). Agricultural management emissions only contributed 5% of total emissions, reflecting cultivation with minimal fertilizer application (Ikazaki et al. 2024). Mitigation efforts should focus on increasing yields through climate‐resilient crop varieties and improved fertilizer management (Haussmann et al. 2012; Weltzien et al. 2018), thus potentially decreasing future LUC. Implementing regulatory targets and financial incentives to encourage the development of enhanced‐efficiency fertilizers is essential (Searchinger et al. 2019). Increasing investment in the agri‐food system, particularly in those improved fertilizer production in developing countries, is crucial to addressing farmers' limited access to locally produced fertilizers (Husmann and Kubik 2019; Jayne and Sanchez 2021). This measure would improve yields while also generating fewer emissions and reducing the necessity to expand into natural vegetation.

The third cluster is characterized by relatively moderate emissions footprints and land requirements, including soybeans in Brazil and rice in SEA. For soybeans, our LUC analysis indicates that 9.8 Mha of natural vegetation was converted to soybean plantations, 36% (3.5 Mha) of which originated from forested areas (tropical forest 0.2 Mha, tropical woodland 1.7 Mha, regrowth forest timber 1.1 Mha, warm mixed forest 0.5 Mha). This conversion resulted in a total of 1.7 GtCO_2_ emissions. In addition, 6 Mha of savanna were converted during 50 years. Soybean expansion peaked in 1990–1995 (Figure 6b), with 1.3 Mha of forest converted, followed by a sharp decline in forest conversion for soybean expansion in the next decades. Studies attribute this decline to more stringent conservation policies, law enforcement, as well as intervention in the agricultural supply chain implemented in the 2000s (Assunção et al. 2015; Nepstad et al. 2014). We observed a decline in both crop footprint and emissions intensity following the introduction of these measures, suggesting the potential influence of policy interventions (see Figure S9). While direct deforestation has decreased since the Soy Moratorium in 2006, soybean expansion continues on previously cleared forestland that has been used for pasture (Macedo et al. 2012). Further reducing emissions from soybeans requires policy interventions in both land‐use management and the agricultural supply chain (Assunção et al. 2015). Rice in SEA represents another case within this cluster, contributing to a total of 6.7 GtCO_2_‐eq. Methane emissions from wetland rice cultivation make up the majority (70%) of these emissions. These methane emissions primarily originate from irrigated and rainfed lowland rice fields, which cover 97% of the region's rice field area (excluding Indonesia) (Bridhikitti and Overcamp 2012). Crop management improvements, such as adopting non‐continuous flooding, can significantly mitigate such emissions from rice cultivation (Bo et al. 2022; Liang et al. 2022).

Emission reduction strategies for other crop‐region pairings can be inferred from our results presented in Table S7 (cumulative) and Table S8 (year 2020). For example, crop‐regions with substantial contributions from drained peatland to cumulative land‐use emissions include cereals in Russia (80%), vegetables in Canada (62%), and palm oil in SEA (40%) and Indonesia (37%). The substantial contribution of drained peatlands in those crop‐regions was also found in previous studies (Mattila 2024; Omar et al. 2022; Strack et al. 2025). Effective mitigation requires a combination of locally appropriate policies and international initiatives focused on restoration, protection, and conservation (Girkin et al. 2022), alongside fostering community‐led stewardship and participation (Mishra et al. 2021; Terzano et al. 2022). Additionally, strengthened monitoring and transparency of corporate activities within plantation concessions that include peatland areas are essential (Evers et al. 2017). We also observed relatively high emissions from agricultural waste burning, particularly for rice and wheat in India and the rest of South Asia. Mitigation strategies include promoting alternative uses of agricultural residues (e.g., power generation or paper production) (Dutta et al. 2022), and at the same time, sustainable crop residue management (i.e., on‐field residue retention) requires careful consideration of soil requirements (Daioglou et al. 2016; Gatkal et al. 2024). Furthermore, encouraging private sector engagement can be supported to connect farmers with the market for waste products (Lin and Begho 2022).

### 3.7 Contributions and Limitations of This Paper

The main novelty of our approach is spatially explicitly attributing land use emissions to agricultural crops and pasture. For LUC emissions, using the coupled IMAGE‐Land and LPJmL model has provided a detailed and accurate representation of carbon density and fluxes. As a result, our analysis offers a more refined assessment of carbon stock changes, thereby improving the estimation of emissions attributed to crops. Furthermore, agricultural management emissions are also modeled within IMAGE using the same underlying land‐use dynamics. This mechanism ensures consistency in the estimation of total land use emissions from both LUC and agricultural management activities, and a comprehensive understanding of agricultural emissions footprints.

Compared to earlier model‐based approaches to attributing emissions to crops, such as bookkeeping methods (Hansis et al. 2015), IMAGE‐LPJmL improves the estimation of carbon density by modeling the actual natural processes. In contrast, bookkeeping models estimate LUC emissions by combining land cover transitions with fixed carbon density values. They track biomass removal resulting only from direct anthropogenic land‐cover change. The bookkeeping approach relies on static parameters, such as fixed carbon densities and predefined decay and regrowth curves for natural vegetation. As a result, it does not capture the effects of climate variability, vegetation dynamics, or CO_2_ fertilization (Poulter et al. 2022). In addition, estimates of agricultural management emissions primarily rely on country‐level data obtained from separate studies or inventories, which may not align with the land‐use dynamics used in the bookkeeping framework. In our study, we incorporate gridded agricultural management emissions modeled within IMAGE to provide a higher level of spatial resolution compared to previous emission attribution studies.

The results presented here go beyond previous analyses by comprehensively including global crop emissions from LUC (all types of natural land) and agricultural management emissions (all key sources of emissions), covering all global crops, and applying finer temporal and spatial granularity. Because we perform a global analysis at the grid level, we capture the heterogeneity of agricultural systems and are able to assess crop emissions that were previously overlooked. In addition, by identifying the types of source emissions dominant in a specific hotspot, a starting point is provided for further analyses aimed at designing specific efforts in reducing the GHG emissions. Although our approach is an improvement compared to other studies in terms of comprehensiveness, this comes with uncertainties. First, the historical agricultural land distribution follows spatial patterns as prescribed by Klein Goldewijk et al. (2011) and calibrated to FAOSTAT. IMAGE allocates agricultural land based on potential crop yields, accessibility, population densities, and terrain slopes from different sources (Doelman et al. 2018). Unlike remote‐sensing datasets that identify specific crop types, IMAGE models land use and crop distribution at an aggregated category level, such as tropical oil crops. Second, the IMAGE model represents land use at a coarse resolution (5′ × 5′ arc min). IMAGE assigns a single dominant land use to each grid cell. However, in reality, a grid most likely contains patches of different land use within its boundaries. Representing approximately 85 km^2^ of land with a single land use category can lead to both under‐ and overestimations of carbon stocks, consequently impacting emission estimates. Third, we applied a 5‐year moving average based on FAOSTAT crop production data to smooth short‐term anomalies and achieve more balanced attribution of emissions. This approach implicitly covers average crop rotation patterns across a region and time, but for specific locations and years, emissions of a specific crop may in some cases be overestimated, meaning others are underestimated.

A limitation of our study is that we could not include the attribution of soil carbon emissions to crops. There are two main reasons for this. First, there are large uncertainties in soil carbon fluxes because changes in soil carbon are typically slower and cumulative over time. Second, there is large complexity in attributing such emissions to crops in a specific year considering that these emissions are caused not only by LUC and agricultural management during that time period but also by previous land use (change). To provide a first‐order estimate of the underestimation from excluding soil carbon pools, we determined soil carbon fluxes of the 5‐year period after the conversion, allocating them directly to crops as we have done for aboveground biomass. The soil carbon emissions share of the total annual LUC emissions is 18%–24% during the respective accounting period (see Figure S10). It is important to note that the initial soil carbon stock of the converted land and subsequent soil carbon dynamics can substantially influence the estimated emissions, their allocation to the first years after conversion, and their relative share of total land use emissions. Focusing only on the first 5 years after conversion is a short‐term approximation of soil carbon changes that should be regarded as a lower‐bound estimation.

## 4 Conclusion and Future Research Avenues

Our study comprehensively addresses land use emissions of all crop types, accounting for both LUC and agricultural management emissions while including their spatio‐temporal variation. Attributing land use emissions to crops is an important step in assessing the emissions of global food supply chains, and it allows identifying crop‐region pairings with high emissions. Looking at three key indicators (absolute emissions, footprints, and intensities) as well as considering trade‐offs between emissions and production, we define three clusters of crop‐region pairings that can help prioritize measures for future reduction in land use emissions from agricultural crops: (i) high footprints, efficient land use (e.g., palm oil in Indonesia) where limiting conversion of high‐carbon stock area has first priority, (ii) low emissions, inefficient land use (e.g., tropical cereals in Western Africa) where focus should be on improved agricultural management to increase yields, thereby also minimizing the need for land expansion, and (iii) moderate footprints and moderate land efficiency (e.g., soybeans in Brazil and rice in Southeast Asia) where a combination of agricultural management improvements and restricting land conversion can help most. Although LUC emissions are dominant globally and preventing further conversion of natural land is key, the increasing emissions from agricultural management imply that additional measures like optimizing synthetic fertilizer use and implementing controlled flooding for rice fields are also essential.

For crop producers, our results provide insights on historic and recent GHG emissions associated with crop production in different regions. From a food company, retailer or consumer perspective, our results allow understanding of crop‐region‐specific emissions footprints, which may ultimately enable end‐consumers to choose greener options. Moreover, widespread understanding of higher emissions of certain products could inspire dietary shifts towards more sustainable consumption among the wider population. The results of our study can also help inform policy makers on which agricultural crops to focus when aiming at reducing greenhouse gas emissions.

Future research avenues include three primary directions. First, it is important to analyze the role of attribution methods and assumptions in the quantification of LUC emissions and their allocation to products (Davis et al. 2014; Hansis et al. 2015). Applying different methodological choices and assumptions to a consistent dataset is crucial to understanding their impact on emission estimates and crop‐level attribution. Second, IMAGE as an IAM framework has the ability to project future agricultural land use and associated emissions under changing climate and socioeconomic scenarios. It offers the opportunity to also assess potential future crop emissions footprints and intensities, identify crop‐region pairings of concern, and study how different mitigation measures affect these. Third, our results on land use emissions attributed to crops can form the foundation for a broader analysis of entire food supply chains and their emissions (Novaes et al. 2017). Integrating spatio‐temporal crop emissions into wider carbon footprint assessments that track commodity trade flows from production to consumption regions allows comprehensively assessing emissions of agricultural value chain actors such as (different kinds of) producers, refineries/processors, intermediate traders, retailers, and even financers, and to improve corporate emission accounting, especially in regard to land use emissions (Hansen et al. 2022). The improved understanding of emissions can, in turn, inform these actors' strategic decisions about sourcing agricultural feedstocks and products, investments, and engagement with value chain actors to reduce emissions.

## Author Contributions

**C. A. Adlan:** conceptualization, data curation, formal analysis, methodology, software, visualization, writing – original draft. **S. V. Hanssen:** conceptualization, methodology, supervision, writing – review and editing. **H. Luchtenbelt:** data curation, methodology, writing – review and editing. **C. Hendriks:** conceptualization, writing – review and editing. **J. C. Doelman:** data curation, writing – review and editing. **E. Stehfest:** data curation, writing – review and editing. **B. Wicke:** conceptualization, funding acquisition, methodology, supervision, writing – review and editing.

## Conflicts of Interest

The authors declare no conflicts of interest.

## Supporting information


**Data S1.** gcb70528‐sup‐0001‐DataS1.pdf.

**Data S2.** gcb70528‐sup‐0001‐DataS2.xlsx.

## Data Availability

The data that support the findings of this study are openly available at https://dataplatform.knmi.nl/organization/pbl and https://www.pbl.nl/en/image/data. The inputs, outputs, and scripts can be found at Dryad (https://doi.org/10.5061/dryad.dz08kps82).
