# Supplementary material for: Emissions Footprints of Agriculture Around the World 1970–2020: Decreasing Land Conversion, Regional Exceptions and Increasing Management Intensity
Source: Glob Chang Biol. 2025 Oct 2;31(10):e70528. doi: 10.1111/gcb.70528 (PMC12491833; doi:10.1111/gcb.70528)
Supplement: Supplementary file 1 — Data S1. gcb70528‐sup‐0001‐DataS1.pdf. [file GCB-31-e70528-s002.pdf]

Supporting Information

Emission footprints of agriculture around the world 1970-2020: decreasing land conversion, regional exceptions and increasing management intensity

C.A. Adlan [a,*], S.V. Hanssen [a], H. Luchtenbelt [b], C. Hendriks [a], J.C. Doelman [b], E. Stehfest [b], B. Wicke [a]

[a] *Department of Environmental Science, Faculty of Science, Radboud University, Nijmegen, The Netherlands*
[b] *PBL Netherlands Environmental Assessment Agency, The Hague, The Netherlands*

* *Corresponding author: chaidir.adlan@ru.nl*

Contents

A. Supporting Methods 3

1. Calculating the carbon stock difference 3
2. Creating transition data and defining transition categories 3
3. Integrating agricultural management emissions 5
4. Attributing land use emissions to agriculture crops 10
5. Generating emissions footprints of crop, crop emission intensities and relationships between emissions and production 10

B. Supporting Figures 11

Figure S1 | IMAGE region classification 11
Figure S2 | Regional land-use transitions over 50 years 12
Figure S3 | Comparison with external studies 19
Figure S4 | Average annual natural vegetation to agriculture emissions per region, per crops 21
Figure S5 | Comparison of the cumulative land use emissions per crop by region 22
Figure S6 | Annual land use emissions over time 24
Figure S7 | Global cumulative land use emissions by region-crop in percent during 1970-2020 with pasture included 25
Figure S8 | Emission intensity comparison 26
Figure S9 | Trends in total emissions footprint and emissions intensity for two selected region-crops, shown alongside the emission reduction policy period 27
Figure S10 | Quantitative estimation from exclusion of soil carbon pools 28

C. Supporting Tables 29

Table S1 | Categorization of land use transitions using land classes from Table SA1 29
Table S2 | Mapping of FAO Crop items to IMAGE Crop Classification 31

Table S3 | Total area converted from natural vegetation to agriculture by origin (Mha)........... 31
Table S4 | Total area converted from natural vegetation to agriculture by crops (Mha) 31
Table S5 | Total area converted from natural vegetation to agriculture by region (Mha).......... 31
Table S6 | Total area converted from natural vegetation to agriculture by natural vegetation category & tropical-temperate regions (Mha) 31
Table S7 | Cumulative land use emission 1970-2020 by crop-region pairing, shares of emissions by emission source and percent share of crop-region pairing in total cumulative global emissions 31
Table S8 | Land use emission year 2020 by crop-region pairing, shares of emissions by emission source and percent share of crop-region pairing in total global emissions year 2020 31
Table S9 | Countries list (with ISO code) that includes in each of 26 regions 32

A. Supporting Methods

1. Calculating the carbon stock difference

We calculate carbon stock loss due to land use transitions in 3 steps (1A – 1B – 1C). Each step is described below.

1A. We converted grid area from kilometre square (km^2) to hectare and carbon density from megagrams (Mg) carbon per kilometre square to tonne carbon per hectare. Three carbon pools in vegetation are included in the calculation: stems, branches and leaves.

$$\boldsymbol{Carbon\ Density_{b,g,t} = Carbon\ Density\_mg_{b,g,t}\ x\ \ 1/km\_to\_ha\ \ [eq.1]}$$
$$\boldsymbol{Carbon\ Stock_{b,g,t} = Carbon\ Density_{b,g,t}\ \ x\ (GAREA_g\ x\ km\_to\_ha)\ [eq.2]}$$
$$\boldsymbol{Carbon\ Stock_{g,t} = \ \sum Carbon\ Stock_{b,g,t}\ [eq.3]}$$

$GAREA_g$	Area (km^2)
$Carbon\ Density_{b,g,t}$	Carbon density in biomass pool *b*, grid *g*, year *t* (ton C/ ha)
km_to_ha	100
$Carbon\ Density\_mg_{b,g,t}$	Carbon density in biomass pool *b*, grid *g*, year *t* (Mg C/ km^2)
$Carbon\ Stock_{b,g,t}$	Total carbon stock in biomass pool *b*, grid *g*, year *t* (ton C)
$Carbon\ Stock_{g,t}$	Total carbon stock in grid *g*, year *t* (ton C)

$t = \{1970, 1975, 1980, 1985, 1990, 1995, 2000, 2005, 2010, 2015, 2020\}$

1B. We quantify the carbon stock difference by applying the stock difference method (IPCC, 2019b).

$$\boldsymbol{CS\ diff_{g,t+1} = Carbon\ Stock_{g,t+1} - \ Carbon\ Stock_{g,t}\ [eq.4]}$$

$CS\ diff_{g,t+1}$	Carbon stock difference in grid *g*, year *t+1* (ton C).

1C. We convert carbon stock difference to carbon emission (Giga ton C)

$$\boldsymbol{C\ Emission_{g,t+1} = CS\ diff_{g,t+1}\ x\ \ {}^{44}/_{12}\ x\ 1e^{-9}\ \ [eq.5]}$$

$C\ Emission_{g,t+1}$	Carbon emission in grid *g*, year *t+1* (Giga ton C).

2. Creating transition data and defining transition categories

For land use change analysis, we define the transitions into emissions or sequestrations event in 4 steps (2A – 2B – 2C – 2D). Each step is described below.

2A. Twenty (20) unique land use classes from IMAGE's Global Land Cover Types (GLCT) dataset (see Table S. A1 below) was used in the analysis. We re-categorized all related natural vegetation as forested or non-forested natural vegetation. Noteworthy is that cropland and intensive pastureland were categorized as agricultural land. Biofuel and extensive grassland remain as single, separate categories.

Table SA1. GLCT Classification (IMAGE's land use classification)

GLCT-nr.	GLCT land use class	Abbrev.	Category
1	agricultural land	agri	agriculture land
2	extensive grassland	extgras	extensive grassland

3	carbon plantation	carb	natveg (non-forested)
4	regrowth forest abandoning	rfab	natveg (forested)
5	regrowth forest timber	rftm	natveg (forested)
6	biofuels	biof	biofuel
7	ice	ice	-
8	tundra	tund	natveg (non-forested)
9	wooded tundra	tuwd	natveg (non-forested)
10	boreal forest	bore	natveg (forested)
11	cool conifer forest	ccfo	natveg (forested)
12	temperate mixed forest	tmfo	natveg (forested)
13	temperate deciduous forest	tdfo	natveg (forested)
14	warm mixed forest	wmfo	natveg (forested)
15	grassland/steppe	stepp	natveg (non-forested)
16	hot desert	dsrt	natveg (non-forested)
17	scrubland	scrb	natveg (non-forested)
18	savanna	svna	natveg (non-forested)
19	tropical woodland	trow	natveg (forested)
20	tropical forest	trof	natveg (forested)

2B. We processed IMAGE's GCLT dataset (20 classes) into a transition dataset that represents transitions from one land use class in the previous to another land use class in the current year (see Table S1).

$$\boldsymbol{Trans_{g,t+1}} = \boldsymbol{LU_{g,t}} \rightarrow \boldsymbol{LU_{g,t+1}} \ \ [\boldsymbol{eq.6}]$$

$\boldsymbol{Trans_{g,t+1}}$	Land use transitions in grid *g*, year *t+1* (ton C)
$\boldsymbol{LU_{g,t}}$	Land use class in grid *g*, year *t*
$\boldsymbol{LU_{g,t+1}}$	Land use class in grid *g*, year *t+1*

2C. The transitions were categorized into six (6) transition categories (see Table S1). The classes are:

- **Natural vegetation transition** is defined as a transition from one natural vegetation to another natural vegetation
- **Natural vegetation to agriculture** is defined as a transition from natural vegetation to managed agricultural land.
- **Natural vegetation to biofuel** is defined as a transition from natural vegetation to biofuels.
- **Natural vegetation to extensive grassland** is defined as a transition from natural vegetation to grassland.
- **Agricultural transition** is a change in the type of managed agricultural land use, where the land remains classified as agricultural, but the crop fraction may change
- **Land Abandonment to natural vegetation** is defined as a transition from agriculture land, extensive grassland, and biofuels to a natural vegetation. Exception: This also includes a land transition where regrowth forest remains as regrowth forest (in sequestration event, see section 2D)

2D. We integrate the transition dataset (step 2C) with the carbon stock difference dataset from step 1C. The datasets $\boldsymbol{Trans_{g,t+1}}$ and $\boldsymbol{C\ Emission_{g,t+1}}$ share identical spatial and temporal

resolutions, hence facilitating seamless integration between the two datasets. Further we are able to distinguish whether the transition is an emission or sequestration event using below conditions:

- The transition category is classified as emission event if $\boldsymbol{C\ Emission_{g,t+1}} > \mathbf{0}$.
- The transition category is classified as sequestration event if $\boldsymbol{C\ Emission_{g,t+1}} < \mathbf{0}$.

In our research we focus on two transitions *"Natural Vegetation to Agriculture"* (LUC – Agri) and *"Agricultural Transitions"* (Agri – trans) as this are the directly relevant for agricultural crop production. The transitions are defined as:

$\boldsymbol{C\ Emission\ LUC - Agri}_{\,g,t+1}$ = $\boldsymbol{C\ Emission_{g,t+1}}$ where $\boldsymbol{LU\ (natveg)}_{\,g,t} \rightarrow \boldsymbol{LU\ (agri)_{g,t+1}}$

$\boldsymbol{C\ Emission\ Agri - trans}_{\,g,t+1}$ = $\boldsymbol{C\ Emission_{g,t+1}}$ where $\boldsymbol{LU\ (agri)}_{\,g,t} \rightarrow \boldsymbol{LU\ (agri)_{g,t+1}}$

3. Integrating agricultural management emissions

We include non-CO_2 LUC emissions and various agricultural practice emissions to our analysis.

3A. There are 7 additional non-CO_2 LUC emissions and both CO_2 and non-CO2 emissions from agricultural management from IMAGE were used in this analysis. Table SA2a includes gridded activity data that we used to derive the emissions. Table SA2b includes gridded emissions data. These datasets have 5-year temporal resolution (moving average) from 1970 to 2020 with 5 arc-minute spatial resolution. We processed all datasets and converted the unit into Gt CO_2-eq.

Table SA2a. Agricultural emissions derived from gridded activity data

Input Dataset	Description	Emission Factor	Uncertainty Range	Unit	Source	Output dataset
GPEATLAND GPEATLAND FRAC	Gridded area of degraded peatland due LUC to crops	See Table SA2c		t C/ha/yr	(IPCC, 2014; Wilson et al., 2016)	Gridded CO_2 emission from degraded peatland
		See Table SA2d		kg CH_4 /ha/yr	(IPCC, 2014; Wilson et al., 2016)	Gridded CH_4 emission from degraded peatland
		See Table SA2e		kg N/ha/yr	(IPCC, 2014; Wilson et al., 2016)	Gridded N_2O emission from degraded peatland
GFERTILIZER	Gridded manure fertilizer application rate (Mg N/km^2 yr)	0.01	0.002 – 0.018	kg N2O–N/kg N	(IPCC, 2019a)	Gridded N_2O emissions from fertilized soils due to manure application
GFERTILIZERSYN	Gridded synthetic fertilizer application rate per crop classes (Mg N/ km^2 yr)	0.01	0.002 – 0.018	kg N2O–N/kg N	(IPCC, 2019a)	Gridded N_2O emissions from synthetic fertilizer application

GABOVERES	Gridded aboveground agriculture residue per crop (Mg dry matter/ km^2)<br><br>See section 3B (equation 7-10)	0.01	0.002 – 0.018	kg N2O–N/kg N	(IPCC, 2019a)	Gridded emission of N_2O from agricultural waste burning
					(IPCC, 2019a)	Gridded emission of CH_4 from agricultural waste burning

Table SA2b. Agricultural emissions derived from gridded activity data

Dataset	Description	Unit	Source
GEN2OLC	Gridded modelled N_2O-N emissions from soil decomposition following deforestation	kg N/km^2/yr	Kreileman & Bouwman, 1994; Stehfest et al., 2014)
GEN2ORE	Gridded modelled N_2O-N emissions from crop residue decomposition<br><br>See section 3C (equation 11)	kg N/km^2 /yr	(JRC/PBL, 2012)
GECH4RI	Gridded modelled CH_4 emissions from wetland rice fields	kg CH4/ km^2/yr	(JRC/PBL, 2012)

Table SA2c. Annual CO_2 emission factor for degraded peatland (t C/ha/yr) is from table 2.1 from IPCC wetland supplement (IPCC, 2014); reclassified to IMAGE agricultural land use categories

Crop type	Boreal	Uncertainty Range (95%CI)	Temperate	Uncertainty Range (95%CI)	Tropical	Uncertainty Range (95%CI)
grass	5.7	2.9 – 8.6	5.9	3.7 – 6.9	9.6	4.5 – 17
wheat	7.9	6.5 – 9.4	7.9	6.5 – 9.4	14.0	6.6 – 26
rice	7.9	6.5 – 9.4	7.9	6.5 – 9.4	9.4	-0.22 – 20
maize	7.9	6.5 – 9.4	7.9	6.5 – 9.4	14.0	6.6 – 26
tropical cereals	7.9	6.5 – 9.4	7.9	6.5 – 9.4	14.0	6.6 – 26
other temperate cereals	7.9	6.5 – 9.4	7.9	6.5 – 9.4	14.0	6.6 – 26
pulses	7.9	6.5 – 9.4	7.9	6.5 – 9.4	14.0	6.6 – 26
soybeans	7.9	6.5 – 9.4	7.9	6.5 – 9.4	14.0	6.6 – 26
temperate oil crops	7.9	6.5 – 9.4	7.9	6.5 – 9.4	14.0	6.6 - 26
tropical oil crops	7.9	6.5 – 9.4	7.9	6.5 – 9.4	15.0	10 - 21
temperate roots & tubers	7.9	6.5 – 9.4	7.9	6.5 – 9.4	14.0	6.6 - 26
tropical roots & tubers	7.9	6.5 – 9.4	7.9	6.5 – 9.4	14.0	6.6 - 26
sugar cane	7.9	6.5 – 9.4	7.9	6.5 – 9.4	14.0	6.6 - 26
oil, palm fruit	7.9	6.5 – 9.4	7.9	6.5 – 9.4	11.0	10 - 21
fruit and vegetables	7.9	6.5 – 9.4	7.9	6.5 – 9.4	14.0	6.6 - 26
other non-food, luxury, spices	7.9	6.5 – 9.4	7.9	6.5 – 9.4	14.0	6.6 - 26
plant based fibres	7.9	6.5 – 9.4	7.9	6.5 – 9.4	14.0	6.6 - 26

Table SA2d. Annual CH_4 emission factor for degraded peatland (kg CH_4/ha/yr) is from table 2.3 from IPCC wetland supplement (IPCC, 2014); reclassified to IMAGE agricultural land use categories

Crop type	Boreal	Uncertainty Range (95%CI)	Temperate	Uncertainty Range (95%CI)	Tropical	Uncertainty Range (95%CI)
grass	1.4	-1.6 – 4.5	18.9	2.4 - 29	7.0	0.3 – 13.7
wheat	0.0	-2.8 – 2.8	0.0	-2.8 – 2.8	7.0	0.3 – 13.7
rice	0.0	-2.8 – 2.8	0.0	-2.8 – 2.8	143.5	63.2 – 223.7
maize	0.0	-2.8 – 2.8	0.0	-2.8 – 2.8	7.0	0.3 – 13.7
tropical cereals	0.0	-2.8 – 2.8	0.0	-2.8 – 2.8	7.0	0.3 – 13.7
other temperate cereals	0.0	-2.8 – 2.8	0.0	-2.8 – 2.8	7.0	0.3 – 13.7
pulses	0.0	-2.8 – 2.8	0.0	-2.8 – 2.8	7.0	0.3 – 13.7
soybeans	0.0	-2.8 – 2.8	0.0	-2.8 – 2.8	7.0	0.3 – 13.7
temperate oil crops	0.0	-2.8 – 2.8	0.0	-2.8 – 2.8	7.0	0.3 – 13.7
tropical oil crops	0.0	-2.8 – 2.8	0.0	-2.8 – 2.8	26.5	7.2 – 45.3
temperate roots & tubers	0.0	-2.8 – 2.8	0.0	-2.8 – 2.8	7.0	0.3 – 13.7
tropical roots & tubers	0.0	-2.8 – 2.8	0.0	-2.8 – 2.8	7.0	0.3 – 13.7
sugar cane	0.0	-2.8 – 2.8	0.0	-2.8 – 2.8	7.0	0.3 – 13.7
oil, palm fruit	0.0	-2.8 – 2.8	0.0	-2.8 – 2.8	0.0	0 – 0
fruit and vegetables	0.0	-2.8 – 2.8	0.0	-2.8 – 2.8	7.0	0.3 – 13.7
other non-food, luxury, spices	0.0	-2.8 – 2.8	0.0	-2.8 – 2.8	7.0	0.3 – 13.7
plant based fibres	0.0	-2.8 – 2.8	0.0	-2.8 – 2.8	7.0	0.3 – 13.7

Table SA2d. Annual N_2O emission factor for degraded peatland (kg N_2O/ha/yr) is from table 2.5 from IPCC wetland supplement (IPCC, 2014); reclassified to IMAGE agricultural land use categories

Crop type	Boreal	Uncertainty Range (95%CI)	Temperate	Uncertainty Range (95%CI)	Tropical	Uncertainty Range (95%CI)
grass	9.50	4.6 – 14	4.70	8.2 – 18	5.0	2.3 – 7.7
wheat	13.0	8.2 – 18	13.0	8.2 – 18	5.0	2.3 – 7.7
rice	13.0	8.2 – 18	13.0	8.2 – 18	0.40	-0.1 – 0.8
maize	13.0	8.2 – 18	13.0	8.2 – 18	5.0	2.3 – 7.7
tropical cereals	13.0	8.2 – 18	13.0	8.2 – 18	5.0	2.3 – 7.7
other temperate cereals	13.0	8.2 – 18	13.0	8.2 – 18	5.0	2.3 – 7.7
pulses	13.0	8.2 – 18	13.0	8.2 – 18	5.0	2.3 – 7.7
soybeans	13.0	8.2 – 18	13.0	8.2 – 18	5.0	2.3 – 7.7
temperate oil crops	13.0	8.2 – 18	13.0	8.2 – 18	5.0	2.3 – 7.7
tropical oil crops	13.0	8.2 – 18	13.0	8.2 – 18	5.0	2.3 – 7.7
temperate roots & tubers	13.0	8.2 – 18	13.0	8.2 – 18	5.0	2.3 – 7.7
tropical roots & tubers	13.0	8.2 – 18	13.0	8.2 – 18	5.0	2.3 – 7.7
sugar cane	13.0	8.2 – 18	13.0	8.2 – 18	5.0	2.3 – 7.7
oil, palm fruit	13.0	8.2 – 18	13.0	8.2 – 18	1.2	n.a.
fruit and vegetables	13.0	8.2 – 18	13.0	8.2 – 18	5.0	2.3 – 7.7
other non-food, luxury, spices	13.0	8.2 – 18	13.0	8.2 – 18	5.0	2.3 – 7.7
plant based fibres	13.0	8.2 – 18	13.0	8.2 – 18	5.0	2.3 – 7.7

3B. We generate CH_4 and N_2O emissions of agricultural waste burning using gridded and crop-specific burned agriculture reside at grid level. It consists of 4 steps as explained below:

3B-1. Generate gridded burned residues per crops

$$\boldsymbol{burnresLoc\_DM_{c,y} = G - abov - res_{c,y} * agwbur_{y,r}\ (eq.7)}$$

$burnresLoc\_DM_{c,y}$	Gridded burned agriculture residue for crop *c*, year *y* (Mg DM/km)
$G - abov - res_{c,y}$	Gridded aboveground agriculture residue for crop *c*, year y (Mg DM/km)
$agwbur_{y,r}$	Fraction of residues burned for year *y*, region *r* (unitless/ fraction)

3B-2. Convert gridded burned residues per crops unit (from Mg DM/km to Mg C/km)

$$\boldsymbol{burnres\_C_{c,y} = burnresLoc\_DM_{c,y} * DM\_to\_C\ \ (eq.8)}$$

$burnres\_C_{c,y}$	Gridded burned agriculture residue for crop *c*, year *y* (Mg C/km)
DM_to_C	Ration dry matter to carbon (unitless/fraction);<br>DM_to_C = 0.48

3B-3. Convert gridded burned residues to emissions

$$\boldsymbol{geCH4ab_{c,y} = burnres\_C_{c,y} * RCH4CAGW\ (eq.9a)}$$

$geCH4ab_{i,y}$	Gridded CH_4 emissions from agriculture waste burning for crop *c*, year *y* (Mg CH_4/ km)
$RCH4CAGW$	Ratio CH_4:C for agricultural waste burning (g CH_4/kg C)<br>RCH4CAGW = 5.63

$$\boldsymbol{geN2Oab_{c,y} = burnres\_C_{c,y} * RN2OCAGW * CNtoN2O\ (eq.9b)}$$

$geN2Oab_{i,y}$	Gridded N_2O emissions from agriculture waste burning for crop *c*, year *y* (Mg N_2O/ km)
$RN2OCAGW$	Ratio N_2O-N:C for agricultural waste burning (g N/kg C)<br>RN2OCAGW = 0.09
$CNtoNO2$	Conversion from N to N20 = 44/28

3B-4. Calculate total emissions per grid

$$\boldsymbol{geCH4ab\_total_{c,y} = geCH4ab_{c,y} * GAREA\ (eq.\ 10a)}$$

$geCH4ab\_total_{c,y}$	Gridded total CH_4 emissions from agriculture waste burning for crop *c*, year *y* (Mg CH_4)
$GAREA$	Grid area (km)

$$\boldsymbol{geN2Oab\_total_{c,y} = geN2Oab_{c,y} * GAREA\ (eq.10b)}$$

$geN2Oab\_total_{c,y}$	Gridded total N_2O emissions from agriculture waste burning for crop *c*, year *y* (Mg N_2O)
$GAREA$	Grid area (km)

3C. Explanation of gridded modelled agricultural emissions

The dataset GEN2OLC represents modeled grid-level N_2O-N emissions (kg N/km^2/yr) arising from following deforestation, as the litter left on the soil surface, root biomass, and soil organic matter decompose, triggering N_2O emissions. To represent this process, emissions during the first year after clearing are assumed to be five times higher than the natural flux of the original forest ecosystem. Thereafter, emissions decrease linearly, reaching the level of the new ecosystem by the tenth year (Stehfest et al., 2014). Model uncertainty arises from the assumption that N_2O emissions occur only in tropical rainforests and seasonal rainforests; The model did not consider N_2O emissions from clearing if other vegetation types such as woods and savannas (Kreileman & Bouwman, 1994)

The dataset GEN2ORE represents modelled grid-level N_2O-N emissions (kg N/km^2/yr) resulting from crop residue decomposition. To estimate the total nitrogen input to soil from crop residues, we first calculate the nitrogen content of aboveground residues remaining on the field after accounting for removals and burning, as well as nitrogen from belowground biomass. For aboveground residues, we determine the amount left on the field by subtracting the fraction of crop residues removed for other uses, such as animal feed, which is modelled by the livestock systems module in IMAGE. Additionally, we subtract the mass of crop residues burned in the field, as this portion does not contribute nitrogen to the soil. The resulting nitrogen input from residues is then multiplied by an emission factor of 0.01 to estimate soil N_2O emissions, expressed as fluxes per unit grid area. The model uncertainty is partly related to the estimation of historical livestock production across different systems, which in turn affects the calculation of the fraction of crop residues removed for other uses, such as animal feed.

Equation 11

$$\boldsymbol{N_{crop_{soil}{}_{c,y}} = N\ from\ aboveground\ crop\ residue\ left\ in\ the\ field + N\ from\ belowground\ residue}$$

$$\boldsymbol{N\_crop\_soil_{c,y} = [(1 - resuse_r)\ x\ (G - abov - res_{c,y} - burnresLoc_{c,y}) * ag\_ncropres_c\ ] + (G - below - res_{c,y} * bg\_ncropres_c)}$$

$$\boldsymbol{N\_crop\_soil_{all\_crops}} = \textbf{sum(}\boldsymbol{N\_crop\_soil_{c,y}}\ \textbf{(crops)}$$

$$\boldsymbol{GENO2RE = \frac{N\_crop\_soil_{all\_crops}}{GAREA_g}\ x\ EN2ORES}$$

$N\_crop\_soil_{c,y}$	Gridded N to soil from agriculture residue for crop *c*, year *y*
$resuse_r$	Fraction of crop residue removed for other uses (animal feed)
$G - abov - res_{c,y}$	Gridded aboveground agriculture residue for crop *c*, year y
$G - below - res_{c,y}$	Gridded belowground agriculture residue for crop *c*, year y
$burnresLoc_{c,y}$	Gridded burned agriculture residue for crop *c*, year *y*
$ag\_ncropres_c$	Nitrogen content of aboveground crop residues for crop *c*.
$bg\_ncropres_c$	Nitrogen content of belowground crop residues for crop *c*.
$EN2ORES$	Emissions factor of direct N_2O emissions from crop residue decomposition (0.01)

The dataset GECH4RI represents modelled grid-level CH_4 emissions (kg CH4/ km^2/yr) resulting from flooded rice fields. The activity data is the area wetland rice, and the emission factor is 22.1 (8.4 – 44.3; min-max range) Tg CH_4/ year (JRC/PBL, 2012) (Doelman et al., 2018). This emission factor is region specific. The uncertainty is coming from estimation of wetland rice area.

4. Attributing land use emissions to agriculture crops

The attribution was conducted at grid level using the seven datasets listed in Table SA2 above. For datasets 1-3, emissions per source were attributed to specific crops using the crop fraction (GFRAC) datasets from IMAGE. Dataset 4 was attributed using gridded burned agriculture residue. Datasets 5–7 were already crop-specific, so no further attribution was required. This attribution of each emissions source to crops resulted in emissions estimates per crop, which were then summed to obtain total land-use emissions per crop (equation 12-14).

$$\textbf{LUC emissions} = \textbf{Natural Vegetation to Agriculture Emissions (per crop)} + \textbf{Agricultural Transistions Emissions (per crop)} + \textbf{GEN2OLC (per crop)} \quad \textit{(eq. 12)}$$

$$\begin{aligned} &\textbf{Agri management emissions (all crops except rice)} \\ &= \textbf{GPEATCO2 (per crop)} + \textbf{GPEATCH4 (per crop)} \\ &+ \textbf{GPEATN2O (per crop)} + \textbf{GFERTILIZER (per crop)} \\ &+ \textbf{GEN2ORE (per crop)} + \textbf{GFERTILIZERSYN (per crop)} \\ &+ \textbf{GEN2OAB (per crop)} + \textbf{GECH4AB (per crop)} \; (\boldsymbol{eq.13a}) \end{aligned}$$

$$\begin{aligned} &\textbf{Agri management emissions (rice only)} = \\ &= \textbf{GPEATCO2 (per crop)} + \textbf{GPEATCH4 (per crop)} \\ &+ \textbf{GPEATN2O (per crop)} + \textbf{GFERTILIZER (per crop)} \\ &+ \textbf{GEN2ORE (per crop)} + \textbf{GFERTILIZERSYN (per crop)} \\ &+ \textbf{GEN2OAB (per crop)} + \textbf{GECH4AB (per crop)} + \textbf{GECH4RI} \; (\boldsymbol{eq.13b}) \end{aligned}$$

$$\textbf{Land use emission} = \textbf{LUC emissions} + \textbf{Agri management emissions} \; (\boldsymbol{eq.14})$$

5. Generating emissions footprints of crop, crop emission intensities and relationships between emissions and production

Emissions footprint of crops and crop emissions intensities were generated at regional level as defined below. The categorization of countries into regional level is based on JRC/PBL (2012).

$$\textbf{Marginal emissions Footprint} = \frac{\textbf{Natural Vegetation to Agriculture Emissions (per crop, sum at regional level)}}{\textbf{Newly converted crop area (per crop, sum at regional level)}} \; (\boldsymbol{eq.15})$$

$$\textbf{Total emissions Footprint Total} = \frac{\textbf{Land use emissions (per crop, sum at regional level)}}{\textbf{Total crop area (per crop, sum at regional level)}} \; (\boldsymbol{eq.16})$$

$$\textbf{Crop emissions intensities} = \frac{\textbf{Land use emissions (per crop, sum at regional level)}}{\textbf{Total crop production (per crop, sum at regional level}} \; (\boldsymbol{eq.17})$$

B. Supporting Figures

Figure S1 | IMAGE region classification

Figure S1 explain 26 IMAGE regions. Countries list (with ISO code) that includes in each of 26 regions with their abbreviations are provided in Table S9.

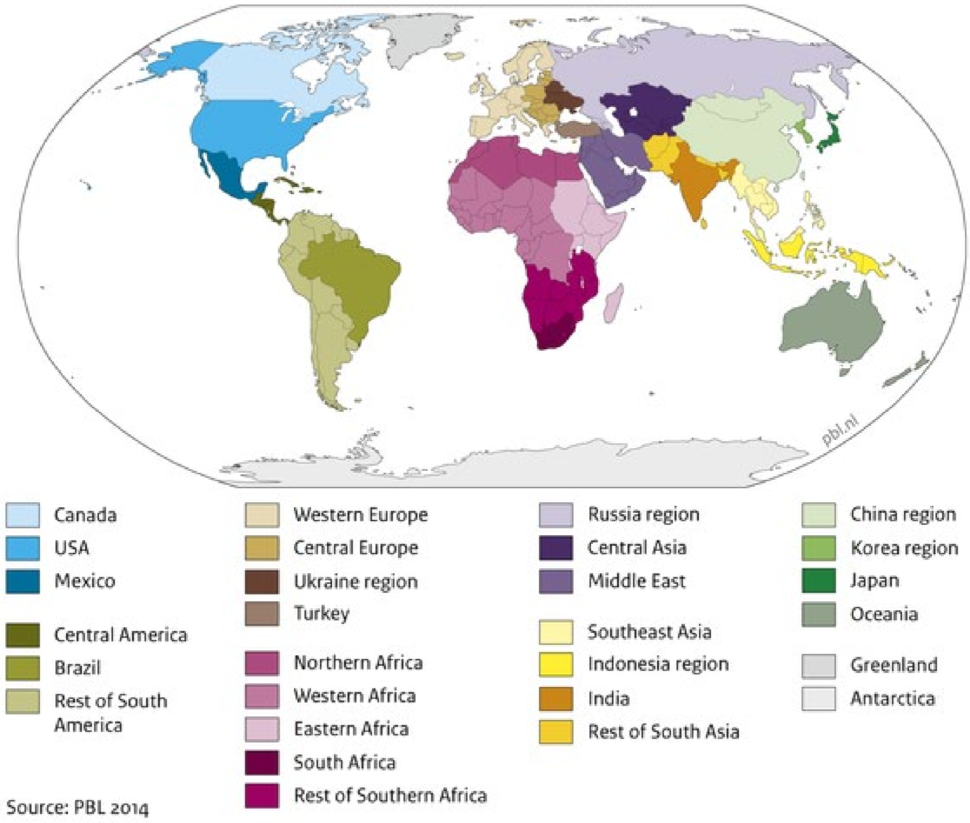

Figure S1. The IMAGE framework region classification (PBL, 2022).

Figure S2 | Regional land-use transitions over 50 years

We generated cumulative land use change patterns for all 26 regions. We focused on conversion of natural vegetation to agricultural land (agricultural land gain), which is the largest source in the LUC emissions category, and land abandonment to natural vegetation (agricultural land loss). We separated converted area originating from forested or non-forested area based on the definition in Table SA1.

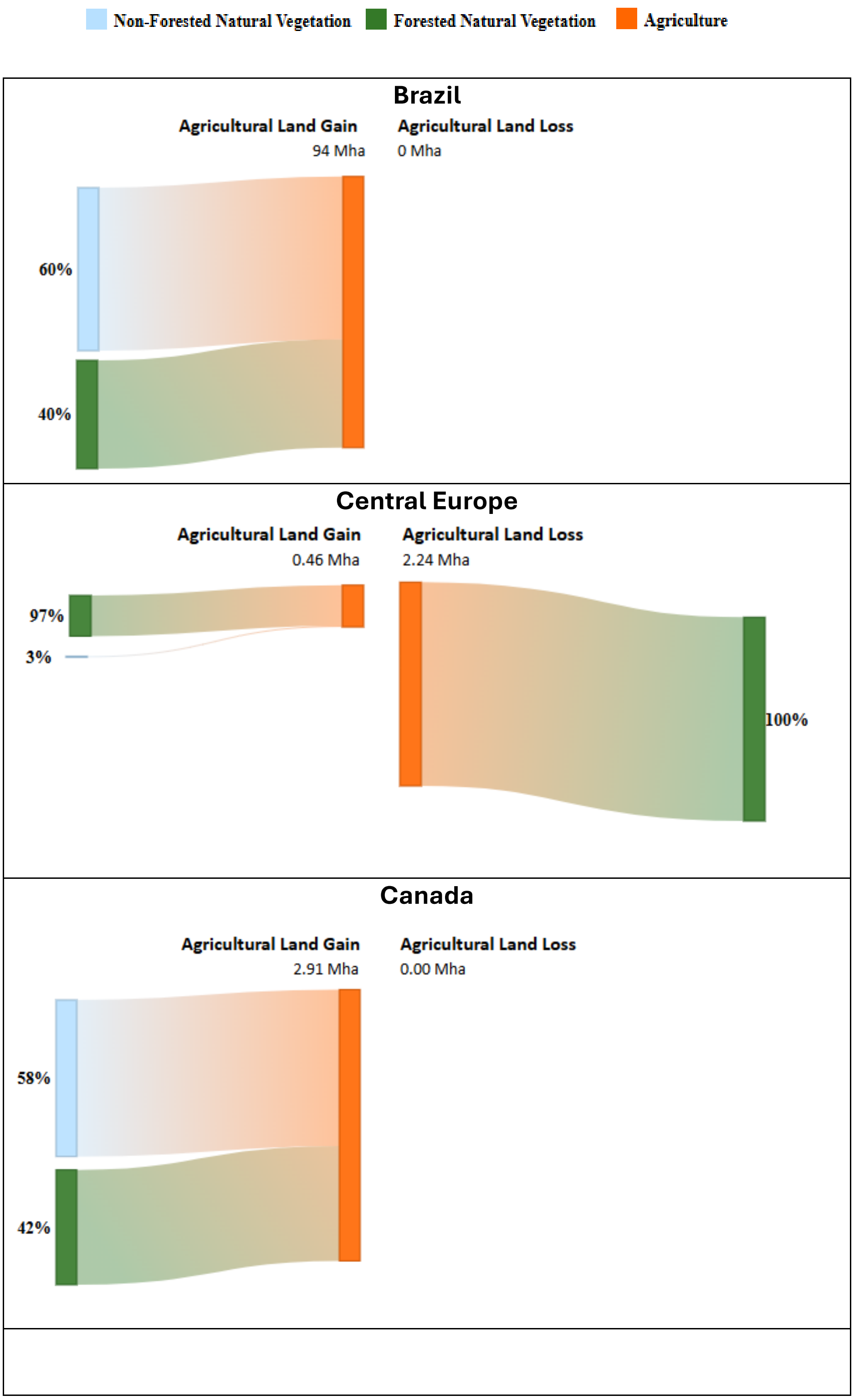

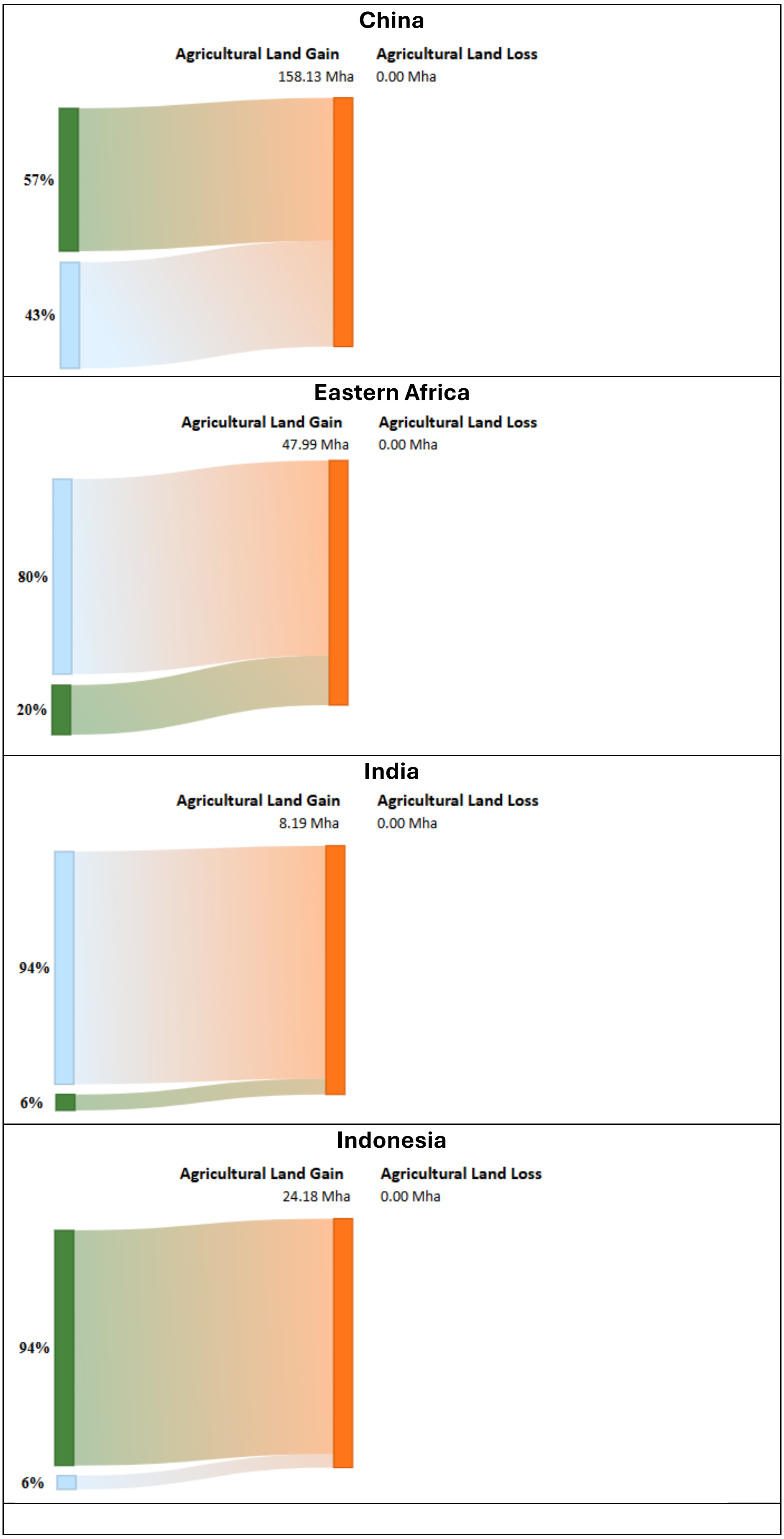

China
Agricultural Land Gain
158.13 Mha
Agricultural Land Loss
0.00 Mha
57%
43%
Eastern Africa
Agricultural Land Gain
47.99 Mha
Agricultural Land Loss
0.00 Mha
80%
20%
India
Agricultural Land Gain
8.19 Mha
Agricultural Land Loss
0.00 Mha
94%
6%
Indonesia
Agricultural Land Gain
24.18 Mha
Agricultural Land Loss
0.00 Mha
94%
6%

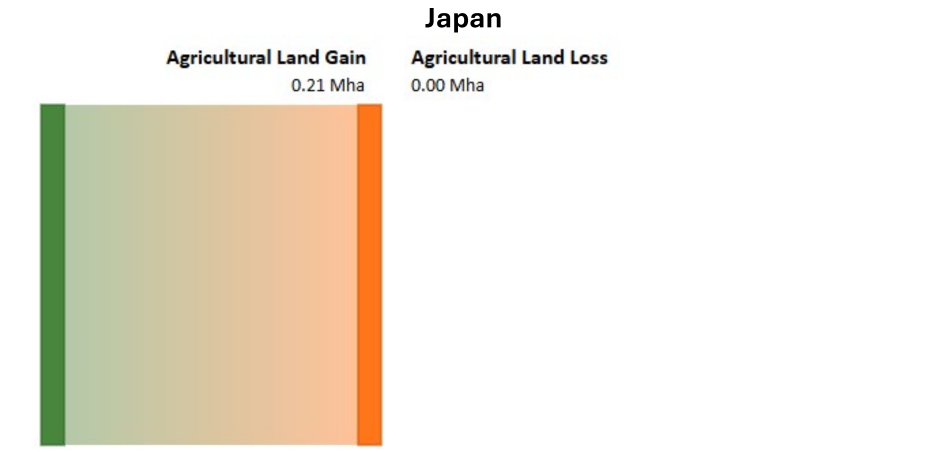
Japan
Agricultural Land Gain
0.21 Mha
Agricultural Land Loss
0.00 Mha

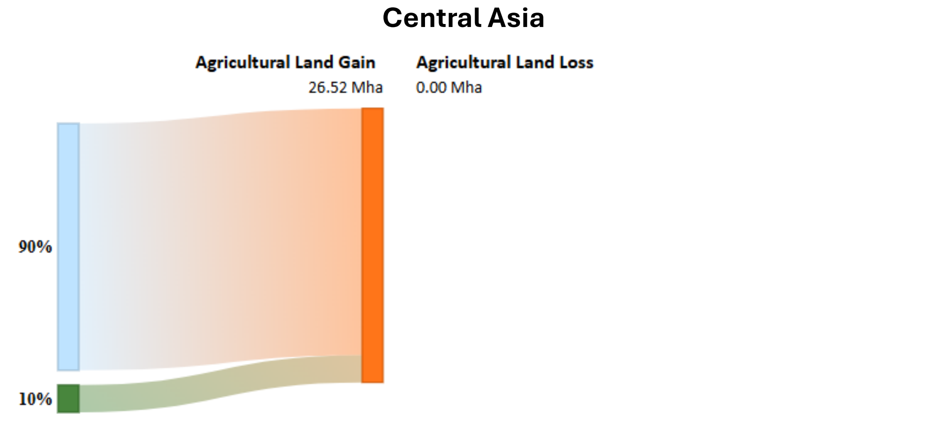
Central Asia
Agricultural Land Gain
26.52 Mha
Agricultural Land Loss
0.00 Mha
90%
10%

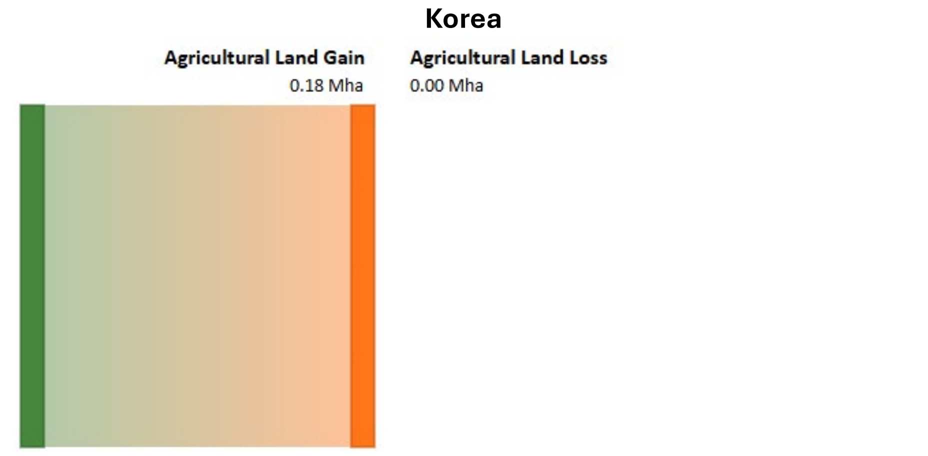
Korea
Agricultural Land Gain
0.18 Mha
Agricultural Land Loss
0.00 Mha

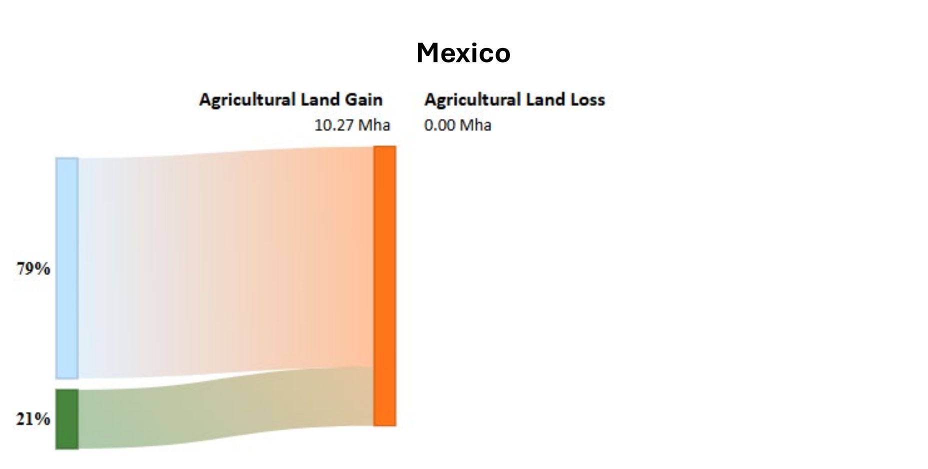
Mexico
Agricultural Land Gain
10.27 Mha
Agricultural Land Loss
0.00 Mha
79%
21%

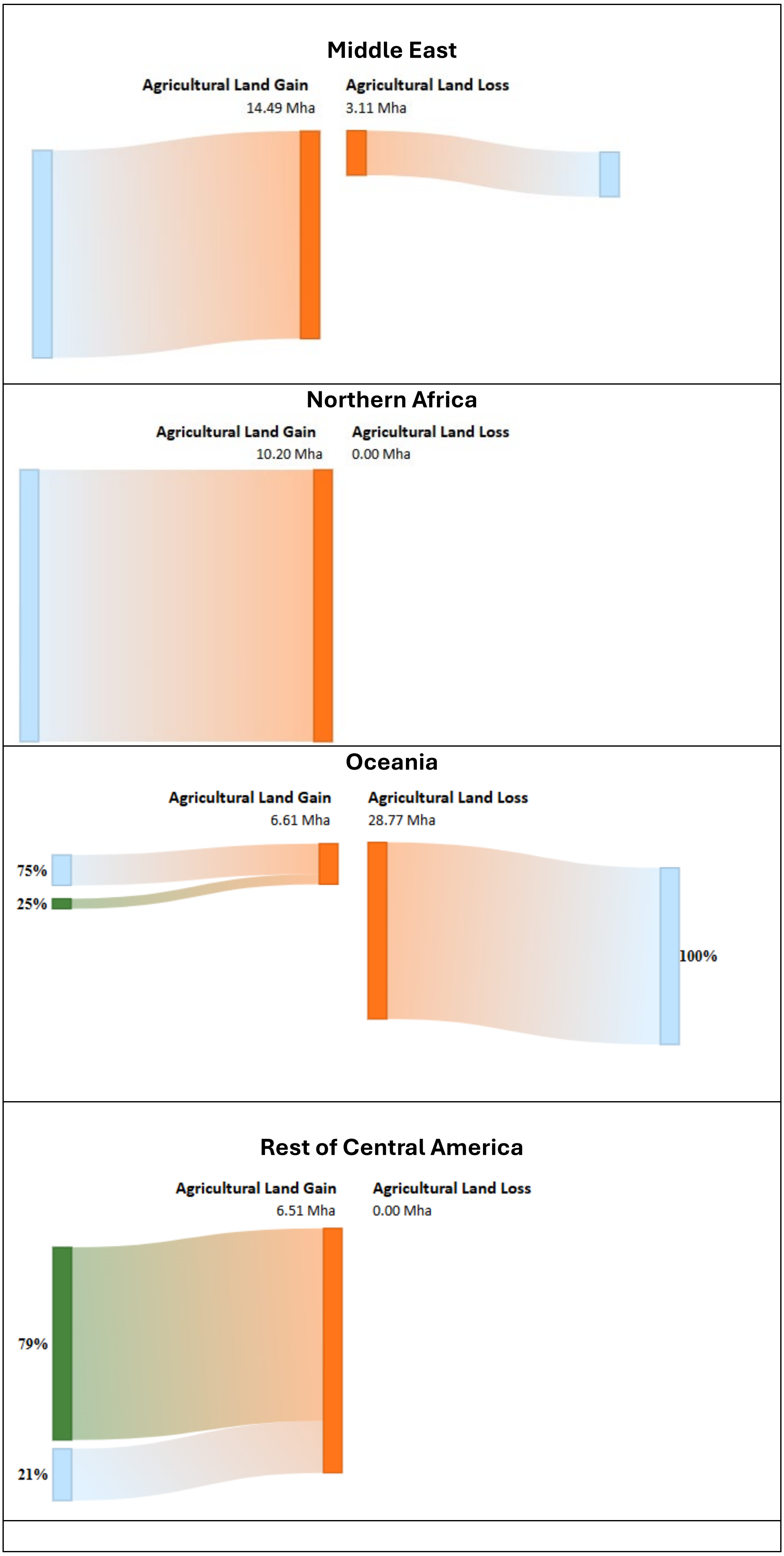
Middle East
Agricultural Land Gain
14.49 Mha
Agricultural Land Loss
3.11 Mha
Northern Africa
Agricultural Land Gain
10.20 Mha
Agricultural Land Loss
0.00 Mha
Oceania
Agricultural Land Gain
6.61 Mha
Agricultural Land Loss
28.77 Mha
75%
25%
100%
Rest of Central America
Agricultural Land Gain
6.51 Mha
Agricultural Land Loss
0.00 Mha
79%
21%

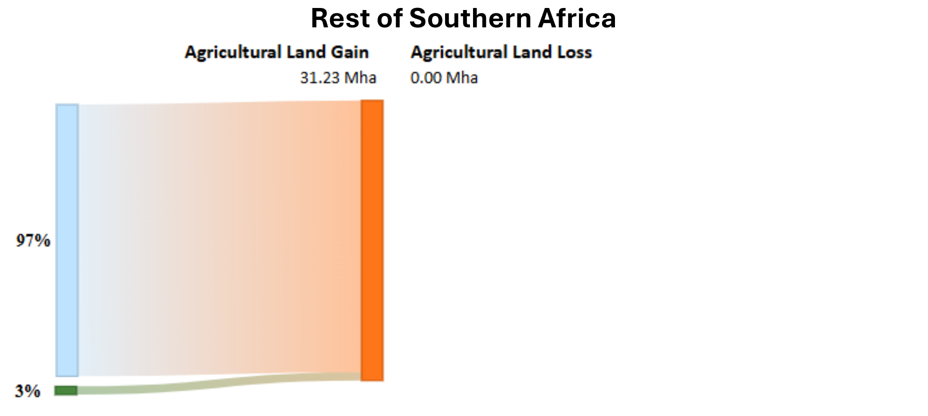
Rest of Southern Africa
Agricultural Land Gain
31.23 Mha
Agricultural Land Loss
0.00 Mha
97%
3%

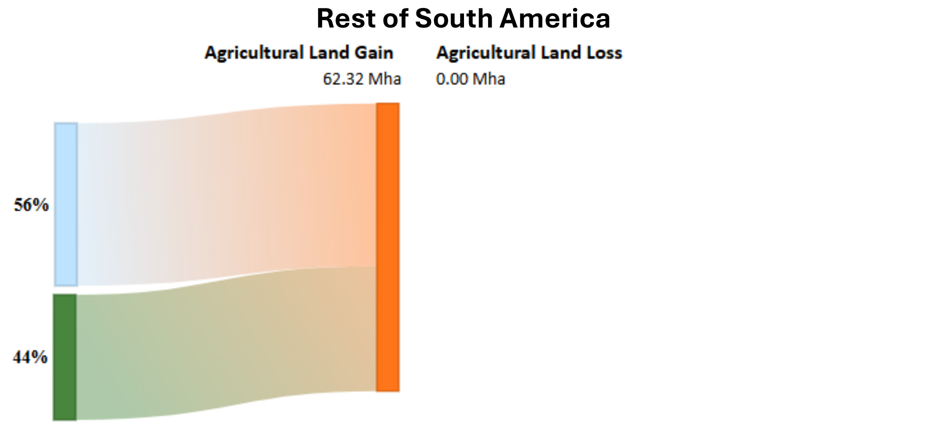
Rest of South America
Agricultural Land Gain
62.32 Mha
Agricultural Land Loss
0.00 Mha
56%
44%

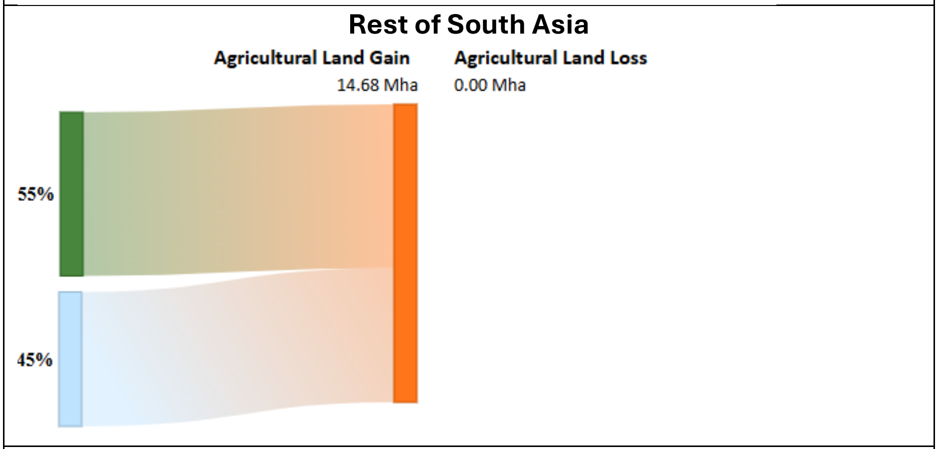
Rest of South Asia
Agricultural Land Gain
14.68 Mha
Agricultural Land Loss
0.00 Mha
55%
45%

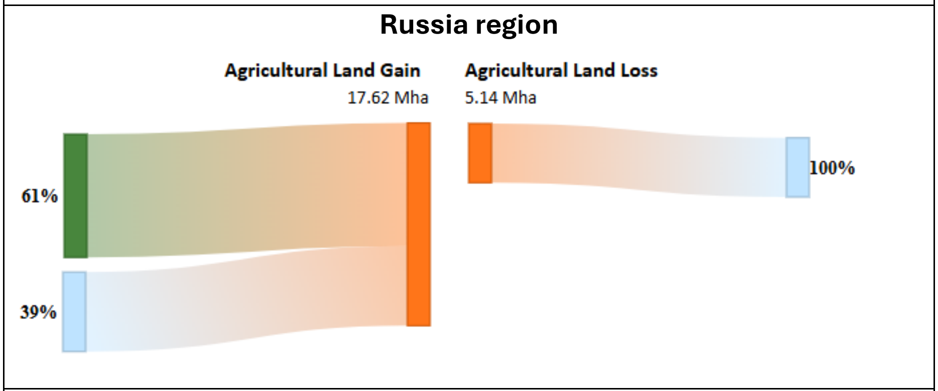
Russia region
Agricultural Land Gain
17.62 Mha
Agricultural Land Loss
5.14 Mha
61%
39%
100%

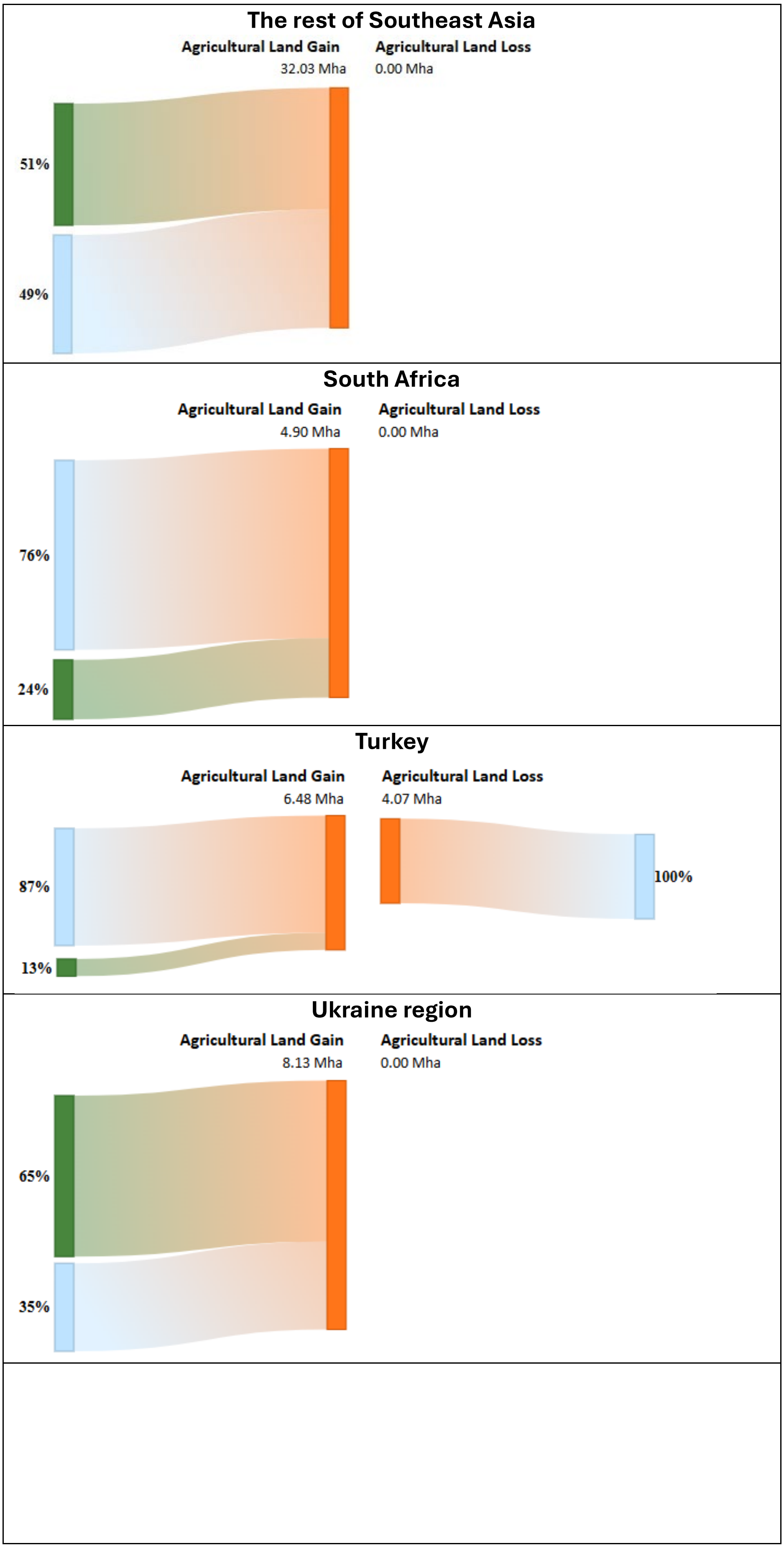

The rest of Southeast Asia
Agricultural Land Gain
32.03 Mha
Agricultural Land Loss
0.00 Mha
51%
49%
South Africa
Agricultural Land Gain
4.90 Mha
Agricultural Land Loss
0.00 Mha
76%
24%
Turkey
Agricultural Land Gain
6.48 Mha
Agricultural Land Loss
4.07 Mha
87%
13%
100%
Ukraine region
Agricultural Land Gain
8.13 Mha
Agricultural Land Loss
0.00 Mha
65%
35%

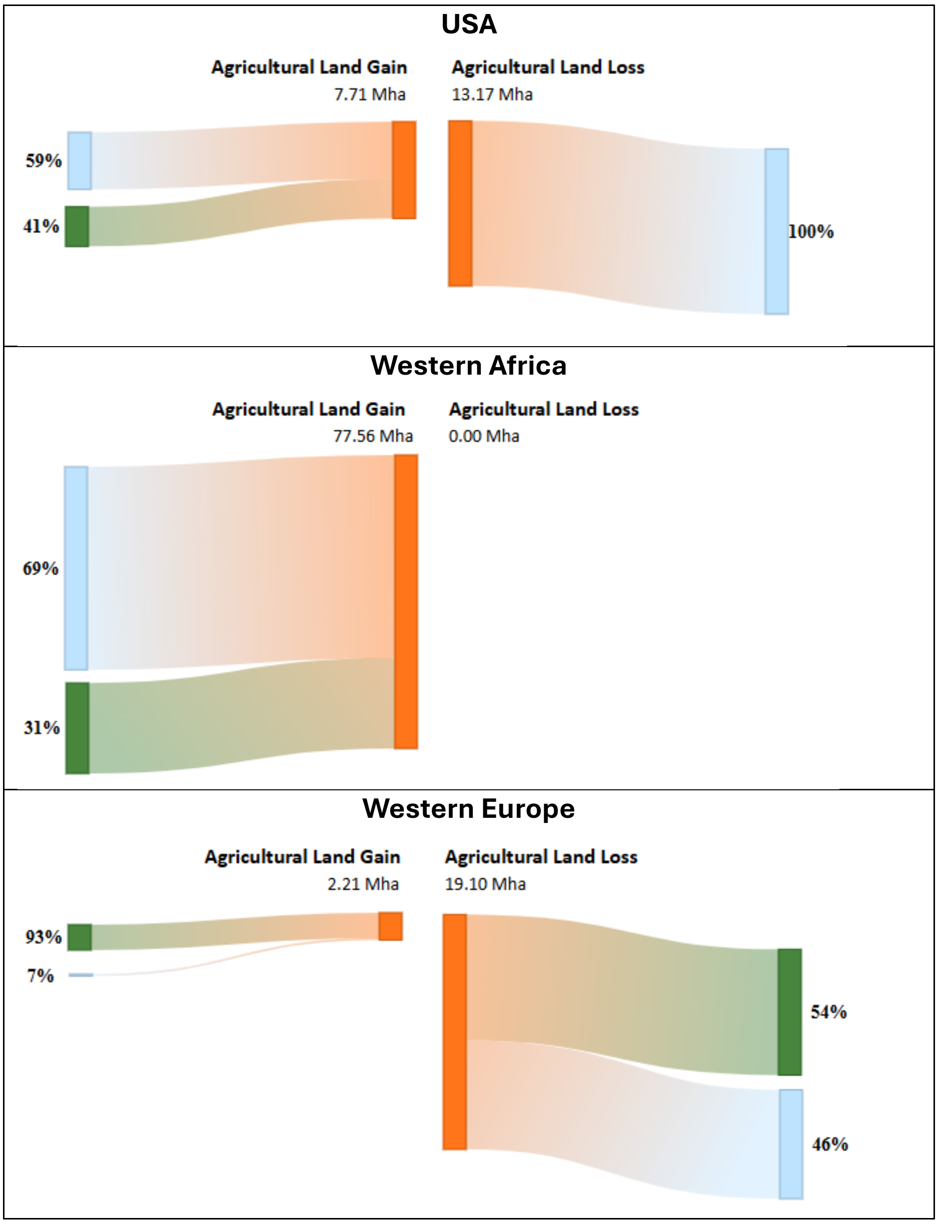

Figure S2. Regional land use transitions over 50 years.

Figure S3 | Comparison with external studies

Here we aim to compare our result with estimates from bookkeeping methods included in Global Carbon Budget 2024 and NGHGI:

a. As our study focuses solely on emissions from the conversion of natural vegetation to agricultural land rather than total net anthropogenic land-use emissions. We compared our estimates with similarly defined emission categories. The Global Carbon Budget 2024 (Friedlingstein et al., 2025) provides disaggregated emissions data from four bookkeeping models (including BLUE, H&C2023, OSCAR, and LUCE datasets (see SI Excel file Global_Carbon_Budget_2024_v1.0). We compare our results with the gross emissions from "deforestation" category from above mentioned dataset, which refers to gross emissions from the conversion of forest to agricultural land (cropland, pasture, or rangeland). To match this definition, we aggregate our emissions for transitions from natural vegetation to cropland and to rangeland. Additionally, we include gross deforestation emissions reported in national greenhouse gas inventories (NGHGI), as compiled in Supplementary Table 4 in Grassi et al. (2021). Our estimates are consistently lower than those estimates but show similar trends. The emissions magnitude differences reflect fundamental methodological differences between process-based models, such as IMAGE-LPJmL, bookkeeping methods, and countries NGHGI report values.

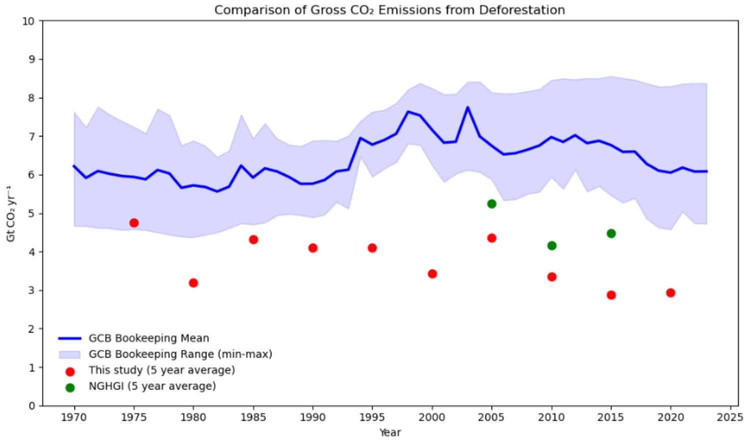

Figure S3a. Comparison of emissions from deforestation estimates

b. For the comparison with NGHGI estimates, it is essential to acknowledge the conceptual differences between IAM-DGVM models (such as IMAGE-LPJmL) and national greenhouse gas inventories (NGHGI) in estimating global anthropogenic net CO_2 emissions from land use. This discrepancy is rooted in a fundamental difference in accounting methodology, as illustrated in Figure 7.6 of the AFOLU chapter, IPCC WG III Sixth Assessment Report (Nabuurs et al., 2022) or Fig. S3b below, based on the analysis by Grassi et al. (2021). Their study found that NGHGI reports are, on average, 5.5 Gt CO_2 yr^{-1} lower than IAM-based estimates, primarily due to differences in system boundaries and definitions (Pongratz et al., 2014), particularly the classification of managed versus unmanaged land (see Fig. 3 in Grassi et al. (2021) as well).

Land-use CO_2 fluxes can be broadly categorized into two types: (1) direct human-induced effects (e.g., land-use change including deforestation, afforestation, harvesting, and

regrowth), which occur only on managed land; and (2) indirect human-induced effects (e.g., CO_2 fertilization, climate change) and natural effects (e.g., interannual climate variability, background disturbance regimes), which affect both managed and unmanaged land (Grassi et al., 2021). NGHGI estimates, following IPCC reporting guidelines, are based on forest inventory data and include only managed lands as a proxy for anthropogenic fluxes. However, these inventories cannot distinguish between direct, indirect, and natural effects, making NGHGI estimates not directly comparable with IAM results unless adjustments are applied (Grassi et al., 2021).

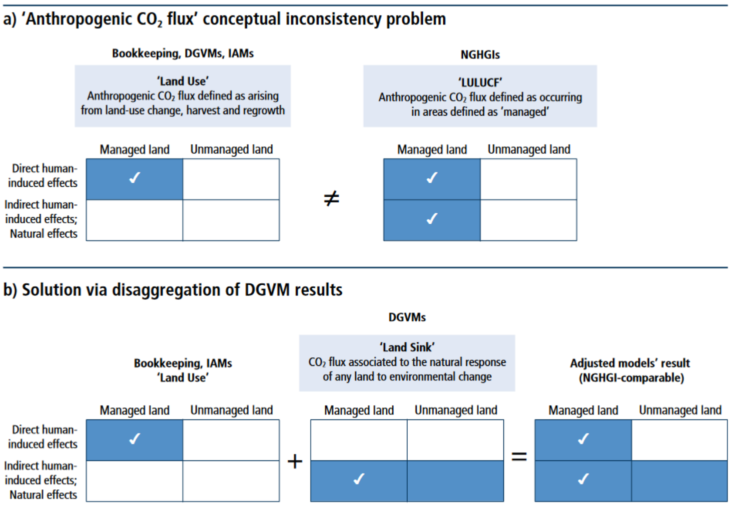

Figure S3b. Conceptual inconsistency problem, the graph was taken from Nabuurs et al. (2022)

Grassi et al. (2021) proposed a harmonization approach to address this issue by disaggregating land carbon sinks attributed to indirect and natural effects into fluxes occurring on managed versus unmanaged forest. This is operationalized by further dividing managed forests into intact and non-intact areas (Hansen et al., 2013; Potapov et al., 2017). Fluxes from indirect effects estimated by DGVMs that occurred in non-intact forests will be added to the IAM estimates. This adjustment allows more consistent comparisons between IAM and NGHGI estimates (see Supplementary Figure 7 in Grassi et al. (2021), for comparison across seven global regions using IMAGE-LPJmL). It is important to emphasize that our study does not apply this adjustment approach. Therefore, the presented IMAGE-LPJmL results remain unadjusted and are not directly comparable with NGHGI reported net emissions values.

Figure S4 | Average annual natural vegetation to agriculture emissions per region, per crops

We attributed only emissions from natural vegetation conversion to agriculture to highlight the largest source of land use emissions, and these results are presented here by crop and region. The exclusion of agricultural management emissions explains the higher pasture shares in Figure S4 compared to Figure 3 in the main text, as several significant emission sources from agricultural management, such as synthetic fertilizer application emissions, were not attributed to pasture.

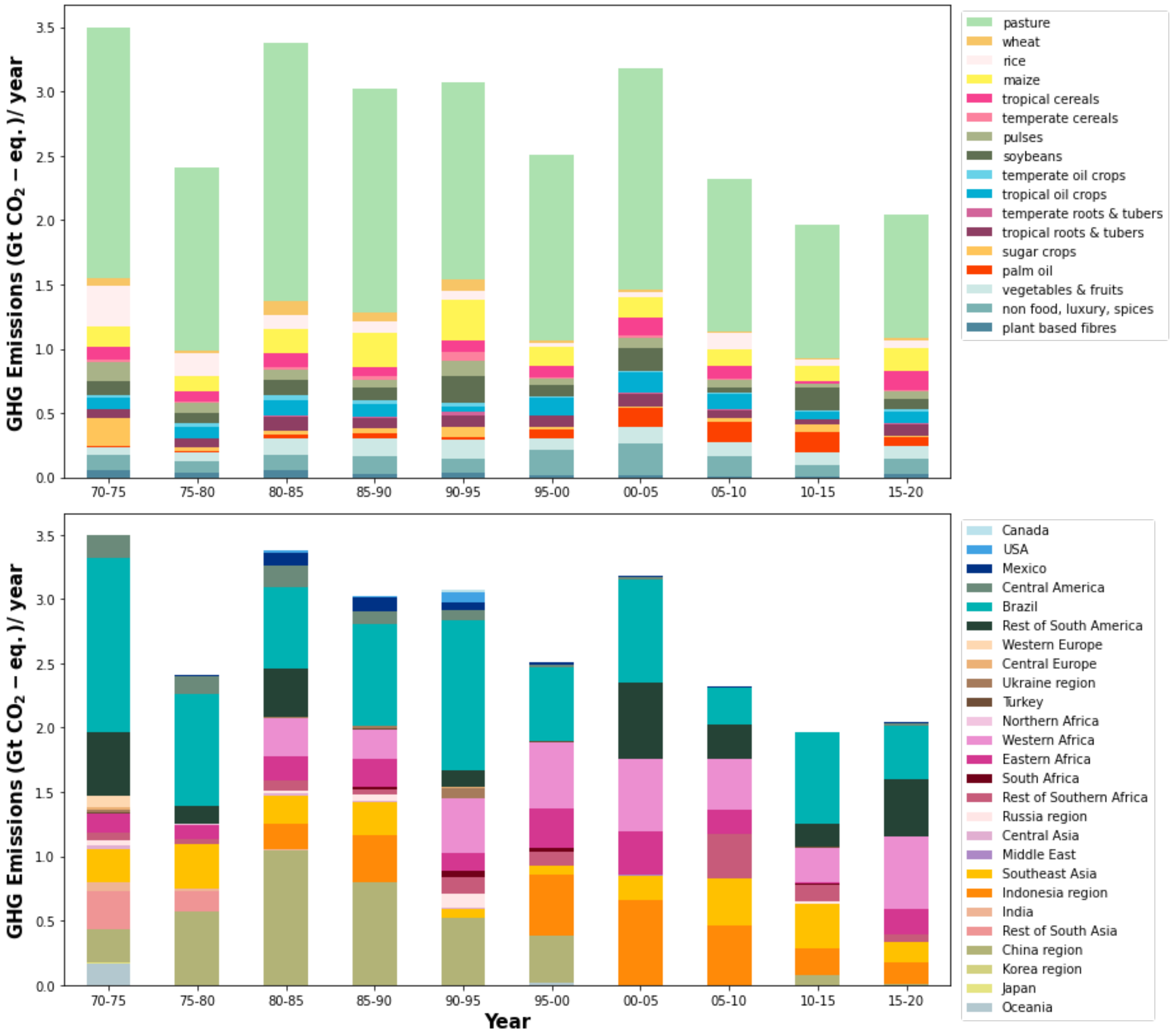

Figure S4. Average annual emissions from conversion of natural vegetation to agriculture a) per crop category, and b) per region

Figure S5 | Comparison of the cumulative land use emissions per crop by region

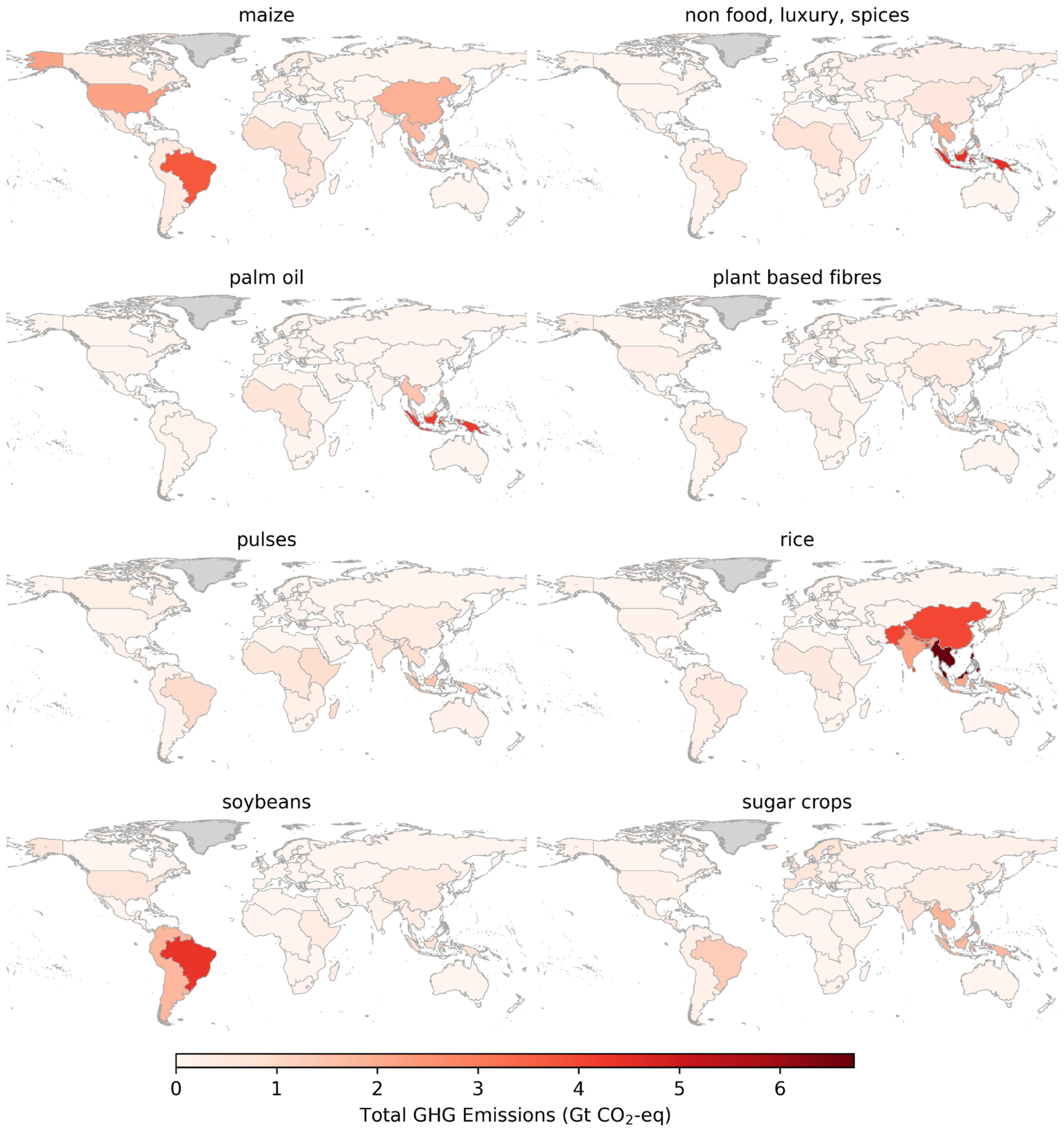

Figure S5a. Cumulative land use emissions per crop category (1)

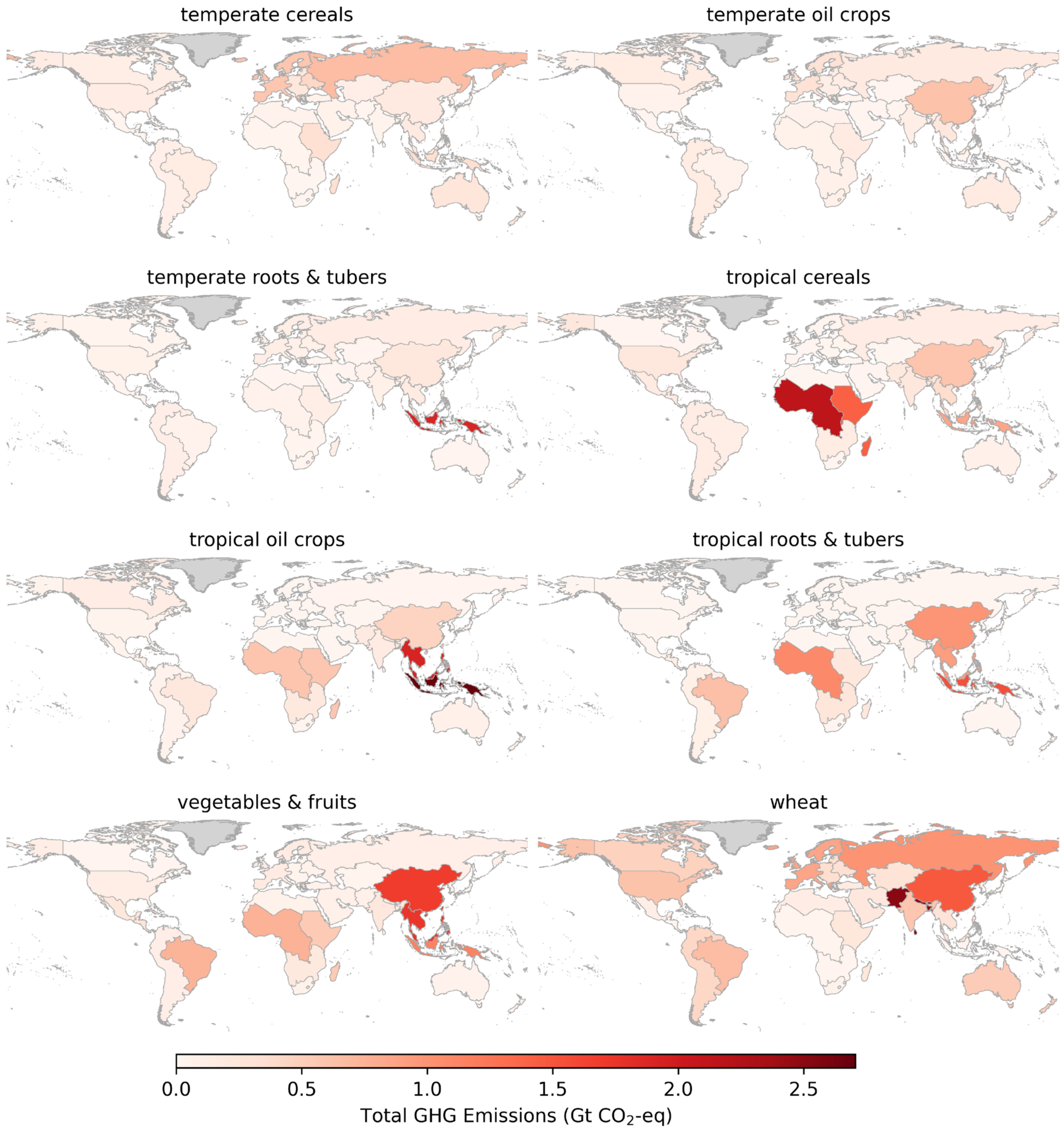

Figure S5b. Cumulative land use emissions per crop category (2)

Figure S6 | Annual land use emissions over time

Figure S6 shows the top three crop-region in their annual land use emissions across the accounting period.

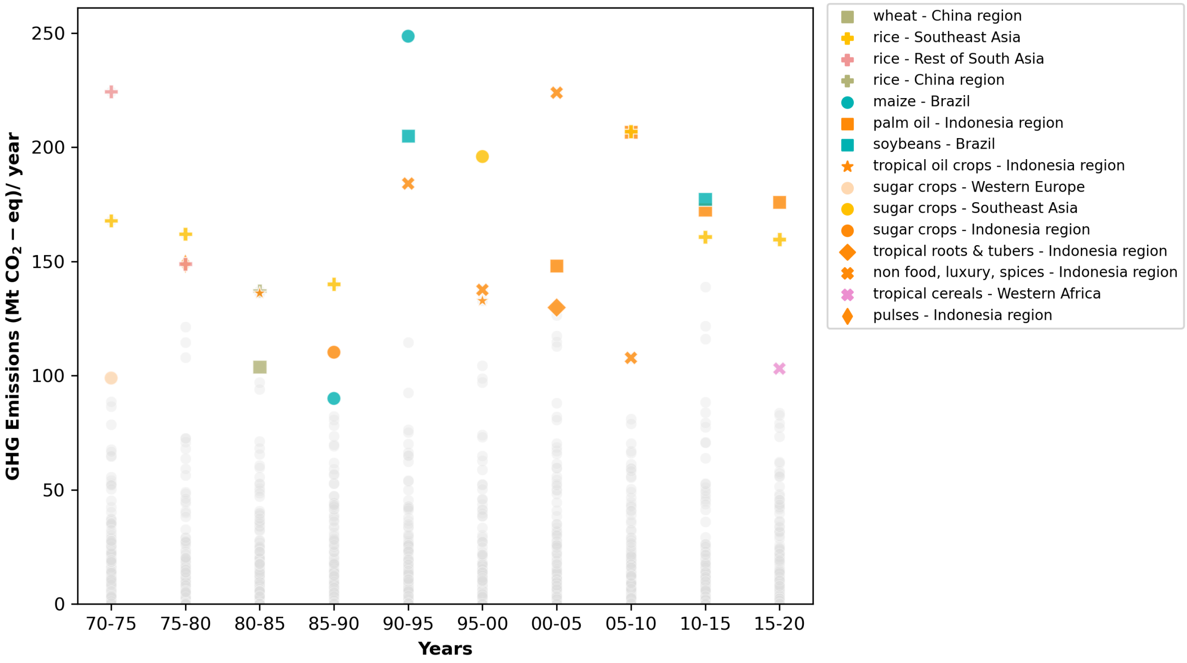

Figure S6. Annual land use emissions (Mt CO_2/ year) over time per crop-region combination, highlighting the top three crop-region combinations per time step

Figure S7 | Global cumulative land use emissions by region-crop in percent during 1970-2020 with pasture included

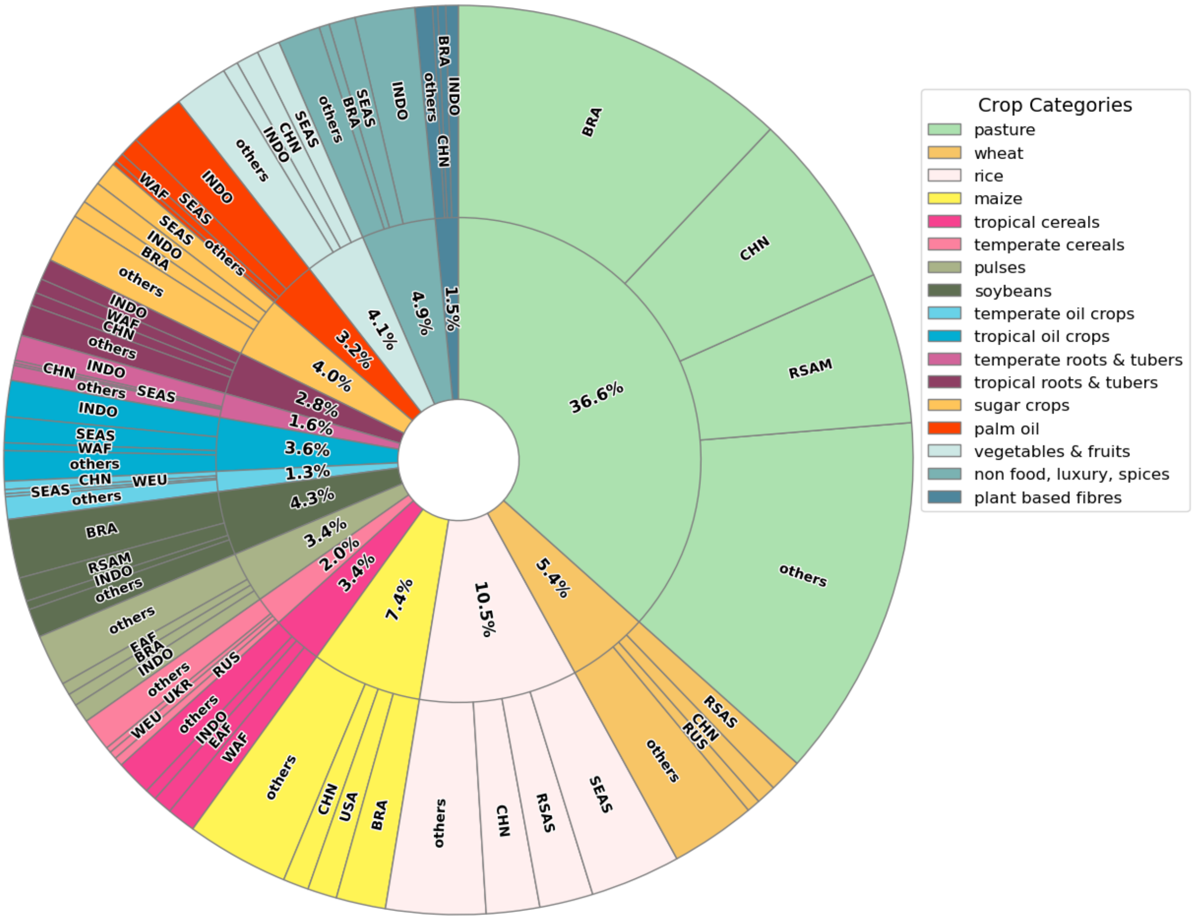

Figure S7. Global cumulative land use emissions by region-crop combination with pasture included

Figure S8 | Emission intensity comparison

We compared our results with values from other comparable studies. For Brazilian soybeans we compared with Escobar et al. (2020); Persson et al. (2014), Indonesia Palm oil with Brinkmann (2009); Flynn et al. (2012); Lam et al. (2021), and Southeast Asia Rice with Arunrat et al. (2021); Taghavi et al. (2017); Wassmann et al. (2022). All studies employed production-based accounting with similar system boundaries, covering land-use change and agricultural management, except for the emissions intensity values for rice, which in selected studies use a broader cradle-to-gate boundary, including machinery use and post-harvest processes, as well as the estimates only from country level in Southeast Asia region.

In this comparison, we recalculated the emissions intensity for palm oil, originally expressed per ton of oil palm fruit in our manuscript, by dividing it by the quantity of palm oil product, to ensure comparability with studies reporting emissions values per ton of palm oil.

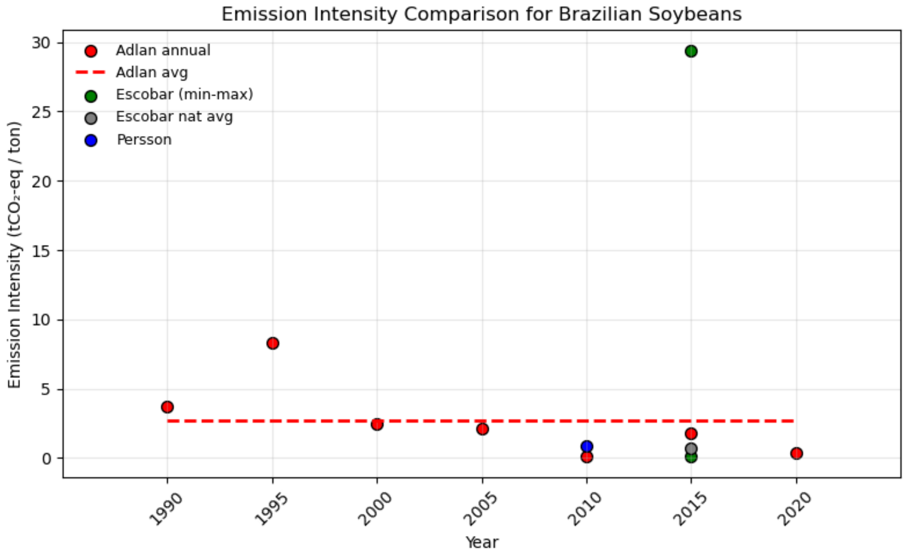

Figure S8a. Emission intensity comparison for Brazilian Soybeans

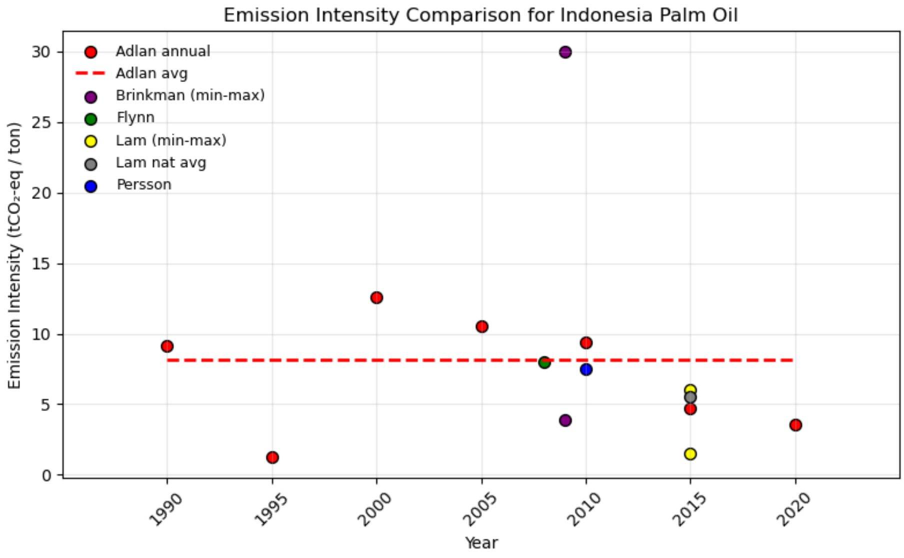

Figure S8b. Emission intensity comparison for Indonesia Palm Oil

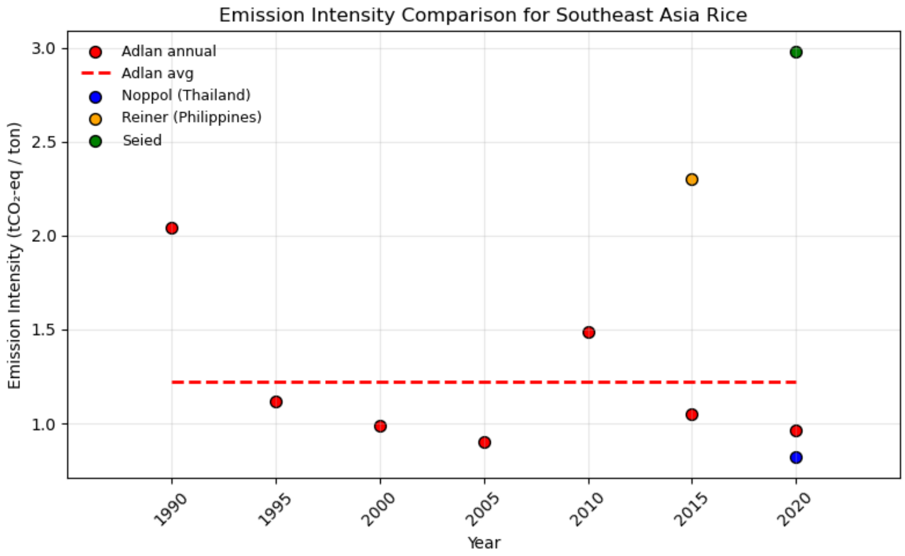

Figure S8c. Emission intensity comparison for Southeast Asia Rice

Figure S9 | Trends in total emissions footprint and emissions intensity for two selected region-crops, shown alongside the emission reduction policy period

Figure S9a show trends in total emissions footprint and emissions intensity for Brazilian soybeans (1995–2020), with the Soy Moratorium initiated in 2006 indicated by the arrow. Nepstad et al. (2014) explained that the Soy Moratorium was not the first measure; several enforcement actions implemented in the early 2000s also contributed to curbing soybean-related emissions. While Figure S9b show trends in total emissions footprint and emissions intensity for Indonesian palm oil (2000–2020), shown alongside the Forest Moratorium policy period. The figure illustrates reductions in both indicators over time, reflecting the potential influence of policy interventions.

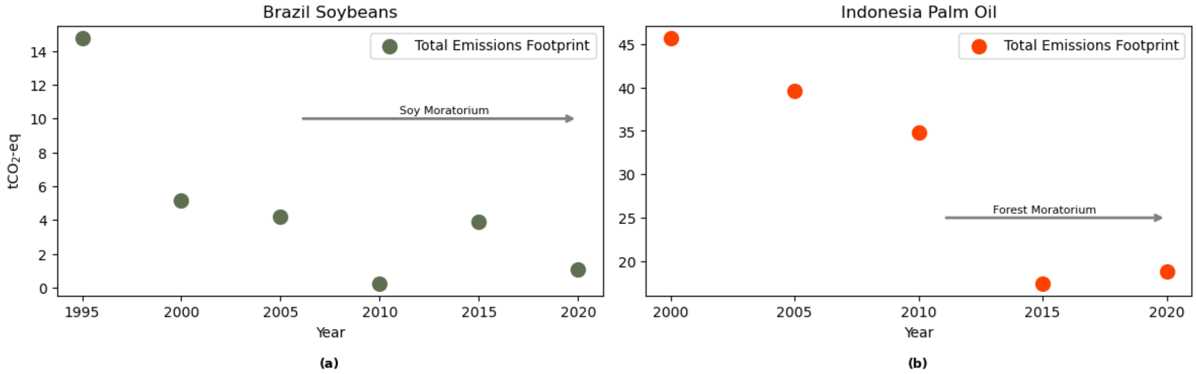

Figure S9. Trend in total emissions footprint (tCO_2-eq/ ha) of (a) Brazilian soybeans (1995-2020) shown alongside Soy Moratorium implemented in 2006 and (b) Indonesian Palm Oil (2000-2020) shown alongside Forest Moratorium implemented in 2011

Figure S10 | Quantitative estimation from exclusion of soil carbon pools

We exclude carbon emissions from disturbed soil carbon pools following land use transitions from natural vegetation to agricultural land. This is due to difficulties in attributing such slower and cumulative emissions to crop in a specific year. In Figure S10 below, we provide quantitative estimation from this exclusion, we determined soil carbon fluxes of the 5-year period after the conversion, allocating them directly to crops as we have done for aboveground biomass. The soil carbon emissions share to the average annual LUC emissions are 18 – 24% during the respective accounting period. It is important to note that the type of land converted, its initial soil carbon stock and subsequent soil carbon dynamics can substantially influence estimated LUC-induced soil carbon emissions, their allocation to the first years after conversion, and their relative share of total land use emissions. Focusing only on the first five years after conversion is a short-term approximation of soil carbon changes that should be regarded as a lower-bound estimation.

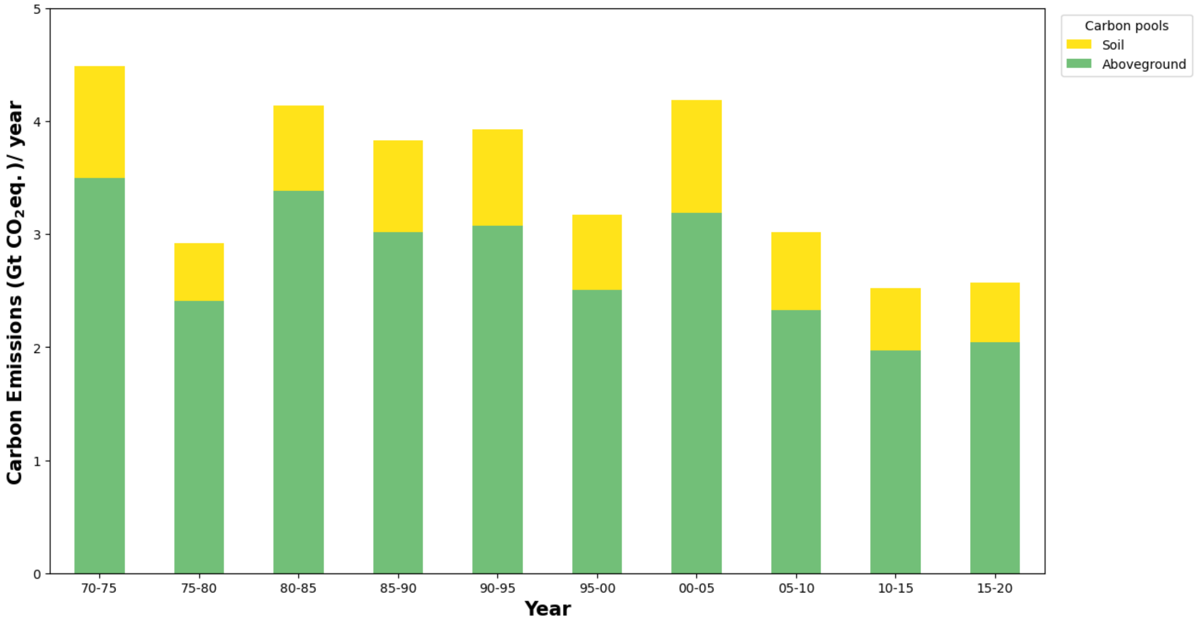

Figure S10. Emission resulted from conversion of natural vegetation to agricultural by including soil carbon pools

C. Supporting Tables

Table S1 | Categorization of land use transitions using land classes from Table SA1.

**Unique transitions**	**General transition category** (If *carbon stock difference* positive then emissions, and if negative then sequestration
agri_to_agri	Agricultural Transition
agri_to_biof	Natural Vegetation to Biofuel
agri_to_dsrt	Land Abandonment
agri_to_rfab	Land Abandonment
agri_to_scrb	Land Abandonment
agri_to_stepp	Land Abandonment
agri_to_svna	Land Abandonment
agri_to_tund	Land Abandonment
agri_to_tuwd	Land Abandonment
biof_to_biof	Natural Vegetation to Biofuel
biof_to_dsrt	Land Abandonment
biof_to_rfab	Land Abandonment
biof_to_scrb	Land Abandonment
biof_to_stepp	Land Abandonment
biof_to_svna	Land Abandonment
biof_to_tund	Land Abandonment
biof_to_tuwd	Land Abandonment
bore_to_agri	Natural Vegetation to Agriculture
bore_to_biof	Natural Vegetation to Biofuel
bore_to_bore	Natural Vegetation transitions
bore_to_rftm	Natural Vegetation transitions
ccfo_to_agri	Natural Vegetation to Agriculture
ccfo_to_biof	Natural Vegetation to Biofuel
ccfo_to_ccfo	Natural Vegetation transitions
ccfo_to_rftm	Natural Vegetation transitions
dsrt_to_agri	Natural Vegetation to Agriculture
dsrt_to_biof	Natural Vegetation to Biofuel
dsrt_to_dsrt	Natural Vegetation transitions
dsrt_to_grass	Natural Vegetation to Extensive Grassland
extgras _to_biof	Natural Vegetation to Extensive Grassland
extgras _to_dsrt	Land Abandonment
extgras _to_ extgras	Natural Vegetation to Extensive Grassland
extgras _to_rfab	Land Abandonment
extgras _to_scrb	Land Abandonment
extgras _to_stepp	Land Abandonment
extgras _to_svna	Land Abandonment
extgras _to_tund	Land Abandonment
extgras _to_tuwd	Land Abandonment
rfab_to_agri	Natural Vegetation to Agriculture
rfab_to_biof	Natural Vegetation to Biofuel
rfab_to_bore	Natural Vegetation transitions
rfab_to_ccfo	Natural Vegetation transitions
rfab_to_rfab	Natural Vegetation transitions
rfab_to_rftm	Natural Vegetation transitions

rfab_to_tdfo	Natural Vegetation transitions
rfab_to_tmfo	Natural Vegetation transitions
rfab_to_trof	Natural Vegetation transitions
rfab_to_trow	Natural Vegetation transitions
rfab_to_wmfo	Natural Vegetation transitions
rftm_to_agri	Natural Vegetation to Agriculture
rftm_to_biof	Natural Vegetation to Biofuel
rftm_to_bore	Natural Vegetation transitions
rftm_to_ccfo	Natural Vegetation transitions
rftm_to_rftm	Natural Vegetation transitions
rftm_to_tdfo	Natural Vegetation transitions
rftm_to_tmfo	Natural Vegetation transitions
rftm_to_trof	Natural Vegetation transitions
rftm_to_trow	Natural Vegetation transitions
rftm_to_wmfo	Natural Vegetation transitions
scrb_to_agri	Natural Vegetation to Agriculture
scrb_to_biof	Natural Vegetation to Biofuel
scrb_to_ extgras	Natural Vegetation to Extensive Grassland
scrb_to_rftm	Natural Vegetation transitions
scrb_to_scrb	Natural Vegetation transitions
stepp_to_agri	Natural Vegetation to Agriculture
stepp_to_biof	Natural Vegetation to Biofuel
stepp_to_ extgras	Natural Vegetation to Extensive Grassland
stepp_to_stepp	Natural Vegetation transitions
svna_to_agri	Natural Vegetation to Agriculture
svna_to_biof	Natural Vegetation to Biofuel
svna_to_rftm	Natural Vegetation transitions
svna_to_svna	Natural Vegetation transitions
tdfo_to_agri	Natural Vegetation to Agriculture
tdfo_to_biof	Natural Vegetation to Biofuel
tdfo_to_rftm	Natural Vegetation transitions
tdfo_to_tdfo	Natural Vegetation transitions
tmfo_to_agri	Natural Vegetation to Agriculture
tmfo_to_biof	Natural Vegetation to Biofuel
tmfo_to_rftm	Natural Vegetation transitions
tmfo_to_tmfo	Natural Vegetation transitions
trof_to_agri	Natural Vegetation to Agriculture
trof_to_biof	Natural Vegetation to Biofuel
trof_to_rftm	Natural Vegetation transitions
trof_to_trof	Natural Vegetation transitions
trow_to_agri	Natural Vegetation to Agriculture
trow_to_biof	Natural Vegetation to Biofuel
trow_to_rftm	Natural Vegetation transitions
trow_to_trow	Natural Vegetation transitions
tund_to_agri	Natural Vegetation to Agriculture
tund_to_biof	Natural Vegetation to Biofuel
tund_to_ extgras	Natural Vegetation to Extensive Grassland
tund_to_rftm	Natural Vegetation transitions
tund_to_tund	Natural Vegetation transitions
tuwd_to_agri	Natural Vegetation to Agriculture
tuwd_to_biof	Natural Vegetation to Biofuel
tuwd_to_ extgras	Natural Vegetation to Extensive Grassland
tuwd_to_rftm	Natural Vegetation transitions

tuwd_to_tuwd	Natural Vegetation transitions
wmfo_to_agri	Natural Vegetation to Agriculture
wmfo_to_biof	Natural Vegetation to Biofuel
wmfo_to_rftm	Natural Vegetation transitions
wmfo_to_wmfo	Natural Vegetation transitions

***There is no agriculture to extensive grassland (extgrass) and vice versa in IMAGE model.*

Table S2 | Mapping of FAO Crop items to IMAGE Crop Classification

(see gcb70528-sup-0002-DataS2.xlsx, sheet name: **FAO_IMAGE_crop**)

Table S3 | Total area converted from natural vegetation to agriculture by origin (Mha)

(see gcb70528-sup-0002-DataS2.xlsx, sheet name: **Natveg_to_AGRI_by_origin**)

Table S4 | Total area converted from natural vegetation to agriculture by crops (Mha)

(see gcb70528-sup-0002-DataS2.xlsx, sheet name: **Natveg_to_AGRI_by_crops**)

Table S5 | Total area converted from natural vegetation to agriculture by region (Mha)

(see gcb70528-sup-0002-DataS2.xlsx, sheet name: **Natveg_to_AGRI_by_region**)

Table S6 | Total area converted from natural vegetation to agriculture by natural vegetation category & tropical-temperate regions (Mha)

(see gcb70528-sup-0002-DataS2.xlsx, sheet name: **Natveg_to_AGRI_by_tropical**)

Table S7 | Cumulative land use emission 1970-2020 by crop-region pairing, shares of emissions by emission source and percent share of crop-region pairing in total cumulative global emissions

(see gcb70528-sup-0002-DataS2.xlsx, sheet name: **cumulative_share_by_sources**)

Table S8 | Land use emission year 2020 by crop-region pairing, shares of emissions by emission source and percent share of crop-region pairing in total global emissions year 2020

(see gcb70528-sup-0002-DataS2.xlsx, sheet name: 2020_share_by_sources)

Table S9 | Countries list (with ISO code) that includes in each of 26 regions

Region (abbr.)	Countries
Canada (CAN)	Canada (124)
USA (USA)	St. Pierre and Miquelon (666), United States (840)
Mexico (MEX)	Mexico (484)
Central America (RCAM)	Anguilla (660), Aruba (533), Bahamas, The (44), Barbados (52), Belize (84), Bermuda (60), Cayman Islands (136), Costa Rica (188), Dominica (212), Dominican Republic (214), El Salvador (222), Grenada (308), Guadeloupe (312), Guatemala (320), Haiti (332), Honduras (340), Jamaica (388), Martinique (474), Montserrat (500), Netherlands Antilles (530), Nicaragua (558), Panama (591), Puerto Rico (630), St. Kitts and Nevis (659), St. Lucia (662), St. Vincent and the Grenadines (670), Trinidad and Tobago (780), Turks and Caicos Isl. (796), Virgin Isl. (Br.) (92), Virgin Islands (U.S.) (850)
Brazil (BRA)	Brazil (76)
Rest of South America (RSAM)	Argentina (32), Bolivia (68), Chile (152), Colombia (170), Ecuador (218), Falklands Isl. (238), French Guyana (254), Guyana (328), Paraguay (600), Peru (604), Suriname (740), Uruguay (858), Venezuela, RB (862)
Northern Africa (NAF)	Algeria (12), Egypt, Arab Rep. (818), Libya (434), Morocco (504), Tunisia (788), Western Sahara (732)
Western Africa (WAF)	Benin (204), Burkina Faso (854), Cameroon (120), Cape Verde (132), Central African Republic (140), Chad (148), Congo, Dem. Rep. (180), Congo, Rep. (178), Cote d'Ivoire (384), Equatorial Guinea (226), Gabon (266), Gambia, The (270), Ghana (288), Guinea (324), Guinea-Bissau (624), Liberia (430), Mali (466), Mauritania (478), Niger (562), Nigeria (566), Sao Tome and Principe (678), Senegal (686), Sierra Leone (694), St. Helena (654), Togo (768)
Eastern Africa (EAF)	Burundi (108), Comoros (174), Djibouti (262), Eritrea (232), Ethiopia (231), Kenya (404), Madagascar (450), Mauritius (480), Reunion (638), Rwanda (646), Seychelles (690), Somalia (706), Sudan (736), Uganda (800)
South Africa (SAF)	South Africa (710)
Western Europe (WEU)	Andorra (20), Austria (40), Belgium (56), Denmark (208), Faeroe Islands (234), Finland (246), France (250), Germany (276), Gibraltar (292), Greece (300), Iceland (352), Ireland (372), Italy (380), Liechtenstein (438), Luxembourg (442), Malta (470), Monaco (492), Netherlands (528), Norway (578), Portugal (620), San Marino (674), Spain (724), Sweden (752), Switzerland (756), United Kingdom (826), Vatican City State (336)
Central Europe (CEU)	Albania (8), Bosnia and Herzegovina (70), Bulgaria (100), Croatia (191), Cyprus (196), Czech Republic (203), Estonia (233), Hungary (348), Latvia (428), Lithuania (440), Macedonia, FYR (807), Poland (616), Romania (642), Serbia and Montenegro (891), Slovak Republic (703), Slovenia (705)
Turkey (TUR)	Turkey (792)
Ukraine region (UKR)	Belarus (112), Moldova (498), Ukraine (804)
Central Asia (STAN)	Kazakhstan (398), Kyrgyz Republic (417), Tajikistan (762), Turkmenistan (795), Uzbekistan (860)

Russia region (RUS)	Armenia (51), Azerbaijan (31), Georgia (268), Russian Federation (643)
Middle East (ME)	Bahrain (48), Iran, Islamic Rep. (364), Iraq (368), Israel (376), Jordan (400), Kuwait (414), Lebanon (422), Oman (512), Qatar (634), Saudi Arabia (682), Syrian Arab Republic (760), United Arab Emirates (784), Yemen, Rep. (887)
India (INDIA)	India (356)
Korea region (KOR)	Korea, Dem. Rep. (408), Korea, Rep. (410)
China region (CHN)	China (156), Hong Kong, China (344), Macao, China (446), Mongolia (496), Taiwan (158)
Southeastern Asia (SEA)	Brunei (96), Cambodia (116), Lao PDR (418), Malaysia (458), Myanmar (104), Philippines (608), Singapore (702), Thailand (764), Vietnam (704)
Indonesia region (INDO)	East Timor (626), Indonesia (360), Papua New Guinea (598)
Japan (JAP)	Japan (392)
Oceania (OCE)	American Samoa (16), Australia (36), Cook Isl. (184), Fiji (242), French Polynesia (258), Kiribati (296), Marshall Islands (584), Micronesia, Fed. Sts. (583), Nauru (520), New Caledonia (540), New Zealand (554), Niue (570), Northern Mariana Islands (580), Palau (585), Pitcairn (612), Samoa (882), Solomon Islands (90), Tokelau (772), Tonga (776), Tuvalu (798), Vanuatu (548), Wallis and Futuna Island (876)
Rest of South Asia (RSAS)	Afghanistan (4), Bangladesh (50), Bhutan (64), Maldives (462), Nepal (524), Pakistan (586), Sri Lanka (144)
Rest of Southern Africa (RSAF)	Angola (24), Botswana (72), Lesotho (426), Malawi (454), Mozambique (508), Namibia (516), Swaziland (748), Tanzania (834), Zambia (894), Zimbabwe (716)

REFERENCES

Arunrat, N., Sereenonchai, S., & Wang, C. (2021). Carbon footprint and predicting the impact of climate change on carbon sequestration ecosystem services of organic rice farming and conventional rice farming: A case study in Phichit province, Thailand. *Journal of Environmental Management*, *289*, 112458. https://doi.org/10.1016/j.jenvman.2021.112458

Brinkmann, A. (2009). *Greenhouse Gas Emissions from Palm Oil Production*. RSPO GHG Working Group.

Doelman, J. C., Stehfest, E., Tabeau, A., van Meijl, H., Lassaletta, L., Gernaat, D. E. H. J., Hermans, K., Harmsen, M., Daioglou, V., Biemans, H., van der Sluis, S., & van Vuuren, D. P. (2018). Exploring SSP land-use dynamics using the IMAGE model: Regional and gridded scenarios of land-use change and land-based climate change mitigation. *Global Environmental Change*, *48*, 119–135. https://doi.org/10.1016/j.gloenvcha.2017.11.014

Escobar, N., Tizado, E. J., zu Ermgassen, E. K. H. J., Löfgren, P., Börner, J., & Godar, J. (2020). Spatially-explicit footprints of agricultural commodities: Mapping carbon emissions embodied in Brazil's soy exports. *Global Environmental Change*, *62*, 102067. https://doi.org/10.1016/j.gloenvcha.2020.102067

Flynn, H. C., Canals, L. M. i., Keller, E., King, H., Sim, S., Hastings, A., Wang, S., & Smith, P. (2012). Quantifying global greenhouse gas emissions from land-use change for crop production. *Global Change Biology*, *18*(5), 1622–1635. https://doi.org/10.1111/j.1365-2486.2011.02618.x

Friedlingstein, P., O'Sullivan, M., Jones, M. W., Andrew, R. M., Hauck, J., Landschützer, P., Le Quéré, C., Li, H., Luijkx, I. T., Olsen, A., Peters, G. P., Peters, W., Pongratz, J., Schwingshackl, C., Sitch, S., Canadell, J. G., Ciais, P., Jackson, R. B., Alin, S. R., ... Zeng, J. (2025). Global Carbon Budget 2024. *Earth System Science Data*, *17*(3), 965–1039. https://doi.org/10.5194/essd-17-965-2025

Grassi, G., Stehfest, E., Rogelj, J., van Vuuren, D., Cescatti, A., House, J., Nabuurs, G.-J., Rossi, S., Alkama, R., Viñas, R. A., Calvin, K., Ceccherini, G., Federici, S., Fujimori, S., Gusti, M., Hasegawa, T., Havlik, P., Humpenöder, F., Korosuo, A., ... Popp, A. (2021). Critical adjustment of land mitigation pathways for assessing countries' climate progress. *Nature Climate Change*, *11*(5), 425–434. https://doi.org/10.1038/s41558-021-01033-6

Hansen, M. C., Potapov, P. V., Moore, R., Hancher, M., Turubanova, S. A., Tyukavina, A., Thau, D., Stehman, S. V., Goetz, S. J., Loveland, T. R., Kommareddy, A., Egorov, A., Chini, L., Justice, C. O., & Townshend, J. R. G. (2013). High-Resolution Global Maps of 21st-Century Forest Cover Change. *Science*, *342*(6160), 850–853. https://doi.org/10.1126/science.1244693

IPCC. (2014). *2013 supplement to the 2006 IPCC guidelines for national greenhouse gas inventories: Wetlands.* . IPCC. https://www.ipcc-nggip.iges.or.jp/public/wetlands/

IPCC. (2019a). *2019 Refinement to the 2006 IPCC Guidelines for National Greenhouse Gas Inventories—Chapter 11*. IPCC. https://www.ipcc-nggip.iges.or.jp/public/2019rf/pdf/4_Volume4/19R_V4_Ch11_Soils_N2O_CO2.pdf

IPCC. (2019b). Chapter 2: Generic methodologies applicable to multiple land-use categories. In: Refinement to the 2006 IPCC Guidelines for National Greenhouse Gas Inventories. In *Generic methodologies applicable to multiple land-use categories* (p. 2.1-2.96). https://www.ipcc-nggip.iges.or.jp/public/2019rf/pdf/4_Volume4/19R_V4_Ch02_Generic%20Methods.pdf

JRC/PBL. (2012). *Emission Database for Global Atmospheric Research (EDGAR).* (Version v4.2 FT2010 ed) [Dataset].

Kreileman, G. J. J., & Bouwman, A. F. (1994). Computing land use emissions of greenhouse gases. *Water, Air, and Soil Pollution*, *76*(1), 231–258. https://doi.org/10.1007/BF00478341

Lam, W. Y., Chatterton, J., Sim, S., Kulak, M., Mendoza Beltran, A., & Huijbregts, M. A. J. (2021). Estimating greenhouse gas emissions from direct land use change due to crop production in multiple countries. *Science of The Total Environment*, *755*, 143338. https://doi.org/10.1016/j.scitotenv.2020.143338

Nabuurs, G. J., Mrabet, R., Hatab, A. A., Bustamante, M., Clark, H., Havlík, P., House, J. I., Mbow, C., Ninan, K. N., Popp, A., Roe, S., Sohngen, B., & Towprayoon, S. (2022). Agriculture, Forestry and Other Land Uses (AFOLU). *Climate Change 2022: Mitigation of Climate Change. Contribution of Working Group III to the Sixth Assessment Report of the Intergovernmental Panel on Climate Change*. https://doi.org/10.1017/9781009157926.009

Nepstad, D., McGrath, D., Stickler, C., Alencar, A., Azevedo, A., Swette, B., Bezerra, T., DiGiano, M., Shimada, J., Seroa da Motta, R., Armijo, E., Castello, L., Brando, P., Hansen, M. C., McGrath-Horn, M., Carvalho, O., & Hess, L. (2014). Slowing Amazon deforestation through public policy and interventions in beef and soy supply chains. *Science*, *344*(6188), 1118–1123. https://doi.org/10.1126/science.1248525

PBL. (2022). *Region classification map—IMAGE*. https://models.pbl.nl/image/Region_classification_map

Persson, U. M., Henders, S., & Cederberg, C. (2014). A method for calculating a land-use change carbon footprint (LUC-CFP) for agricultural commodities – applications to Brazilian beef and soy, Indonesian palm oil. *Global Change Biology*, *20*(11), 3482–3491. https://doi.org/10.1111/gcb.12635

Pongratz, J., Reick, C. H., Houghton, R. A., & House, J. I. (2014). Terminology as a key uncertainty in net land use and land cover change carbon flux estimates. *Earth Syst. Dynam*, *5*, 177–195. https://doi.org/10.5194/esd-5-177-2014

Potapov, P., Hansen, M. C., Laestadius, L., Turubanova, S., Yaroshenko, A., Thies, C., Smith, W., Zhuravleva, I., Komarova, A., Minnemeyer, S., & Esipova, E. (2017). The last frontiers of wilderness: Tracking loss of intact forest landscapes from 2000 to 2013. *Science Advances*, *3*(1), e1600821. https://doi.org/10.1126/sciadv.1600821

Stehfest, E., van Vuuren, D., Kram, T., & Bouwman, L. (2014). *IMAGE 3.0 Model description and policy applications*. https://www.pbl.nl/sites/default/files/downloads/PBL-2014-Integrated_Assessment_of_Global_Environmental_Change_with_IMAGE_30-735_1.pdf

Taghavi, S. M., Mendoza, T. C., Acero Jr, B., Li, T., Siddiq, S. A., Yorobe Jr, J., Li, Z., & Ali, J. (2017). Carbon Dioxide Equivalent Emissions of Newly Developed Rice Varieties. *Journal of Agricultural Science*, *9*(5), 107. https://doi.org/10.5539/jas.v9n5p107

Wassmann, R., Van-Hung, N., Yen, B. T., Gummert, M., Nelson, K. M., Gheewala, S. H., & Sander, B. O. (2022). Carbon Footprint Calculator Customized for Rice Products: Concept and Characterization of Rice Value Chains in Southeast Asia. *Sustainability*, *14*(1), Article 1. https://doi.org/10.3390/su14010315

Wilson, D., Blain, D., & Couwenberg, J. (2016). Greenhouse gas emission factors associated with rewetting of organic soils. *Mires and Peat*, *17*, 1–28. https://doi.org/10.19189/MaP.2016.OMB.222
